# Supplementary material for: Association between hypertension and self-perception of health status: Findings from a decade population-based survey in Spanish adults
Source: PLoS One. 2025 May 7;20(5):e0322577. doi: 10.1371/journal.pone.0322577 (PMC12057956; doi:10.1371/journal.pone.0322577)
Supplement: Appendix 1 — (DOCX) [file pone.0322577.s001.docx]

**Appendix 1. Final explanative model**

**Stat code:** gologit2 (varlist) [pweight = weighting], vce(cluster Region) autofit lrforce

**Results:**

Testing the proportional odds assumption using the .05 level of significance.

Step 1: Constraints for parallel lines imposed for Regular physical activity (Yes vs. No) (P Value = 0.8552)

Step 2: Constraints for parallel lines imposed for Mental disorder (Yes vs. No) (P Value = 0.5535)

Step 3: Constraints for parallel lines imposed for Year (P Value = 0.3481)

Step 4: Constraints for parallel lines imposed for Marital status (Married vs. Not married) (P Value = 0.0632)

Step 5: Proportionality constraints are not imposed for

Gender (*P*-value = 0.02127)

Age (*P*-value < 0.00001)

HTN (Yes vs. No) (*P*-value = 0.00044)

Medication for HTN (Yes vs. No) (*P*-value = 0.00003)

Limitation (Yes vs. No) (*P*-value < 0.00001)

Cardiovascular disease other than HTN (Yes vs. No) (*P*-value < 0.00001)

Osteoarticular pathology (Yes vs. No) (*P*-value < 0.00001)

Chronic respiratory disease (Yes vs. No) (*P*-value < 0.00001)

Recent anxiety/depression (Yes vs. No) (*P*-value < 0.00001)

Polypharmacy (Yes vs. No) (*P-value < 0.00001*)

Current smoker (Yes vs. No) (*P*-value = 0.02220)

Normal weight (Yes vs. No) (*P*-value = 0.00001)

High social class (High vs rest) (*P*-value < 0.00001)

Generalised ordered Logit estimates Number of observations = 61,933

Wald chi2(14) = 44,458.09 Prob > chi2 < 0.0001

Log pseudolikelihood = -58,882.539 Pseudo R2 = 0.2260

| **1 vs 2+3+4+5** | Coef | SE | Z | *p*>z | ILCI95% | SLCI% |
| --- | --- | --- | --- | --- | --- | --- |
| Year | -0.14529 | 0.06384 | -2.28000 | 0.02300 | -0.27042 | -0.02016 |
| Women vs. men | 0.06839 | 0.03712 | 1.84000 | 0.06500 | -0.00436 | 0.14113 |
| Age (years) | 0.02568 | 0.00140 | 18.38000 | <0.00001 | 0.02294 | 0.02842 |
| Married vs. rest | 0.06718 | 0.02152 | 3.12000 | 0.00200 | 0.02500 | 0.10935 |
| HTN vs. rest | 0.42601 | 0.10934 | 3.90000 | <0.00001 | 0.21170 | 0.64032 |
| HTN Medication vs. rest | 0.31069 | 0.09432 | 3.29000 | 0.00100 | 0.12583 | 0.49555 |
| Limitation vs. rest | 1.72734 | 0.06128 | 28.19000 | <0.00001 | 1.60722 | 1.84745 |
| Other CV disease vs. rest | 0.61779 | 0.09872 | 6.26000 | <0.00001 | 0.42430 | 0.81127 |
| Osteoarticular pathology vs. rest | 0.96821 | 0.12929 | 7.49000 | <0.00001 | 0.71481 | 1.22161 |
| Chronic respiratory disease vs. rest | 0.58927 | 0.04900 | 12.03000 | <0.00001 | 0.49323 | 0.68531 |
| Mental disorder vs. rest | 0.53923 | 0.05825 | 9.26000 | <0.00001 | 0.42507 | 0.65340 |
| Current anxiety/depression vs. rest | 0.65239 | 0.09305 | 7.01000 | <0.00001 | 0.47002 | 0.83476 |
| Polypharmacy vs. rest | 0.36988 | 0.19247 | 1.92000 | 0.05500 | -0.00735 | 0.74711 |
| Current smoker vs. rest | 0.18579 | 0.03786 | 4.91000 | <0.00001 | 0.11159 | 0.25999 |
| Physical activity vs. rest | -0.40805 | 0.05674 | -7.19000 | <0.00001 | -0.51926 | -0.29684 |
| Normal weight vs. rest | -0.20104 | 0.02443 | -8.23000 | <0.00001 | -0.24892 | -0.15317 |
| High social class vs. rest | -0.30114 | 0.03431 | -8.78000 | <0.00001 | -0.36838 | -0.23390 |
| Constant | -0.03212 | 0.19406 | -0.17000 | 0.86900 | -0.41247 | 0.34822 |
|  |  |  |  |  |  |  |
| **1+2 vs 3+4+5** | Coef | SE | Z | *p*>z | ILCI95% | SLCI95% |
| Year | -0.14529 | 0.06384 | -2.28000 | 0.02300 | -0.27042 | -0.02016 |
| Women vs. men | 0.14019 | 0.02363 | 5.93000 | <0.00001 | 0.09387 | 0.18650 |
| Age (years) | 0.01900 | 0.00112 | 16.97000 | <0.00001 | 0.01681 | 0.02120 |
| Married vs. rest | 0.06718 | 0.02152 | 3.12000 | 0.00200 | 0.02500 | 0.10935 |
| HTN vs. rest | 0.31263 | 0.07266 | 4.30000 | <0.00001 | 0.17021 | 0.45505 |
| A HTN H Medication vs. rest | 0.10414 | 0.07117 | 1.46000 | 0.14300 | -0.03535 | 0.24362 |
| Limitation vs. rest | 2.07030 | 0.04267 | 48.52000 | <0.00001 | 1.98667 | 2.15393 |
| Other CV disease vs. rest | 0.59334 | 0.05542 | 10.71000 | <0.00001 | 0.48471 | 0.70196 |
| Osteoarticular pathology vs. rest | 0.84116 | 0.03825 | 21.99000 | <0.00001 | 0.76619 | 0.91613 |
| Chronic respiratory disease vs. rest | 0.68614 | 0.03971 | 17.28000 | <0.00001 | 0.60830 | 0.76397 |
| Chronic mental disorder vs. rest | 0.53923 | 0.05825 | 9.26000 | <0.00001 | 0.42507 | 0.65340 |
| Current anxiety/depression vs. rest | 0.83546 | 0.05666 | 14.75000 | <0.00001 | 0.72441 | 0.94650 |
| Polypharmacy vs. rest | 0.77077 | 0.05527 | 13.94000 | <0.00001 | 0.66244 | 0.87911 |
| Current smoker vs. rest | 0.06393 | 0.04216 | 1.52000 | 0.12900 | -0.01870 | 0.14655 |
| Physical activity vs. rest | -0.40805 | 0.05674 | -7.19000 | <0.00001 | -0.51926 | -0.29684 |
| Normal weight vs. rest | -0.15809 | 0.01583 | -9.99000 | <0.00001 | -0.18912 | -0.12706 |
| High social class vs. rest | -0.45390 | 0.01401 | -32.40000 | <0.00001 | -0.48136 | -0.42644 |
| Constant | -3.00980 | 0.12941 | -23.26000 | <0.00001 | -3.26344 | -2.75616 |
|  |  |  |  |  |  |  |
|  |  |  |  |  |  |  |
| **1+2+3 vs 4+5** | Coef | SE | Z | *p*>z | ILCI95% | SLCI95% |
| Year | -0.14529 | 0.06384 | -2.28000 | 0.02300 | -0.27042 | -0.02016 |
| Women vs. men | 0.01779 | 0.04232 | 0.42000 | 0.67400 | -0.06516 | 0.10075 |
| Age (years) | 0.01788 | 0.00266 | 6.72000 | <0.00001 | 0.01267 | 0.02309 |
| Married vs. rest | 0.06718 | 0.02152 | 3.12000 | 0.00200 | 0.02500 | 0.10935 |
| HTN vs. rest | 0.10904 | 0.06895 | 1.58000 | 0.11400 | -0.02610 | 0.24418 |
| HTN Medication vs. rest | -0.06251 | 0.05233 | -1.19000 | 0.23200 | -0.16508 | 0.04006 |
| Limitation vs. rest | 2.57353 | 0.09786 | 26.30000 | <0.00001 | 2.38173 | 2.76534 |
| Other CV disease vs. rest | 0.42467 | 0.04537 | 9.36000 | <0.00001 | 0.33575 | 0.51358 |
| Osteoarticular pathology vs. rest | 0.46502 | 0.04139 | 11.24000 | <0.00001 | 0.38390 | 0.54613 |
| Chronic respiratory disease vs. rest | 0.38154 | 0.06088 | 6.27000 | <0.00001 | 0.26221 | 0.50086 |
| Mental disorder vs. rest | 0.53923 | 0.05825 | 9.26000 | <0.00001 | 0.42507 | 0.65340 |
| Current anxiety/depression vs. rest | 0.52152 | 0.05555 | 9.39000 | <0.00001 | 0.41264 | 0.63041 |
| Polypharmacy vs. rest | 0.56916 | 0.07797 | 7.30000 | <0.00001 | 0.41634 | 0.72198 |
| Current smoker vs. rest | 0.14461 | 0.07832 | 1.85000 | 0.06500 | -0.00889 | 0.29811 |
| Physical activity vs. rest | -0.40805 | 0.05674 | -7.19000 | <0.00001 | -0.51926 | -0.29684 |
| Normal weight vs. rest | -0.01171 | 0.06085 | -0.19000 | 0.84700 | -0.13097 | 0.10756 |
| High social class vs. rest | -0.30728 | 0.05584 | -5.50000 | <0.00001 | -0.41671 | -0.19784 |
| Constant | -5.20437 | 0.13904 | -37.43000 | <0.00001 | -5.47688 | -4.93186 |
| **1+2+3+4 vs 5** | Coef | SE | Z | *p*>z | ILCI95% | SLCI95% |
| Year | -0.14529 | 0.06384 | -2.28000 | 0.02300 | -0.27042 | -0.02016 |
| Women vs. men | 0.08743 | 0.05779 | 1.51000 | 0.13000 | -0.02584 | 0.20070 |
| Age (years) | 0.01383 | 0.00461 | 3.00000 | 0.00300 | 0.00480 | 0.02286 |
| Married vs. rest | 0.06718 | 0.02152 | 3.12000 | 0.00200 | 0.02500 | 0.10935 |
| HTN vs. rest | -0.03399 | 0.07771 | -0.44000 | 0.66200 | -0.18629 | 0.11831 |
| HTN Medication vs. rest | 0.14638 | 0.10714 | 1.37000 | 0.17200 | -0.06360 | 0.35637 |
| Limitation vs. rest | 3.22440 | 0.19733 | 16.34000 | <0.00001 | 2.83764 | 3.61116 |
| Other CV disease vs rest | 0.47679 | 0.07677 | 6.21000 | <0.00001 | 0.32633 | 0.62724 |
| Osteoarticular pathology vs. rest | 0.24387 | 0.07442 | 3.28000 | 0.00100 | 0.09801 | 0.38974 |
| Chronic respiratory disease vs. rest | 0.23629 | 0.06580 | 3.59000 | <0.00001 | 0.10733 | 0.36524 |
| Mental disorder vs. rest | 0.53923 | 0.05825 | 9.26000 | <0.00001 | 0.42507 | 0.65340 |
| Current anxiety/depression vs. rest | 0.46504 | 0.15706 | 2.96000 | 0.00300 | 0.15721 | 0.77287 |
| Polypharmacy vs. rest | 0.45366 | 0.11719 | 3.87000 | <0.00001 | 0.22396 | 0.68335 |
| Current smoker vs. rest | 0.29068 | 0.12972 | 2.24000 | 0.02500 | 0.03644 | 0.54492 |
| Physical activity vs. rest | -0.40805 | 0.05674 | -7.19000 | <0.00001 | -0.51926 | -0.29684 |
| Normal weight vs. rest | 0.11711 | 0.07853 | 1.49000 | 0.13600 | -0.03680 | 0.27102 |
| High social class vs. rest | -0.02833 | 0.09413 | -0.30000 | 0.76300 | -0.21282 | 0.15615 |
| Constant | -7.54053 | 0.29573 | -25.50000 | <0.00001 | -8.12014 | -6.96092 |

HTN: arterial hypertension; CV: cardiovascular; SE: Standard Error; IL/SL CI95%. Inferior/Superior limit of the 95% confidence interval

Perception of health status: 1 “very good”, 2 “good”; 3 “fair”; 4 “bad”; 5 “very bad”.
