# Supplementary material for: Association between hypertension and self-perception of health status: Findings from a decade population-based survey in Spanish adults
Source: PLoS One. 2025 May 7;20(5):e0322577. doi: 10.1371/journal.pone.0322577 (PMC12057956; doi:10.1371/journal.pone.0322577)
Supplement: Appendix 2 — (DOCX) [file pone.0322577.s002.docx]

**Appendix 2. Final explanative model. Marginal effects**

**Stata code:** margins, dydx(*)

| **predict(pr outcome(1))** | dy/dx | SE | Z | *p*>z | ILCI95% | SLCI95% |
| --- | --- | --- | --- | --- | --- | --- |
| Year | 0.0212 | 0.0092 | 2.2900 | 0.0220 | 0.0030 | 0.0393 |
| Women vs. men | -0.0100 | 0.0055 | -1.8200 | 0.0690 | -0.0207 | 0.0008 |
| Age (years) | -0.0037 | 0.0002 | -15.7200 | 0.0000 | -0.0042 | -0.0033 |
| Married vs. rest | -0.0098 | 0.0031 | -3.1300 | 0.0020 | -0.0159 | -0.0037 |
| HTN vs. rest | -0.0620 | 0.0158 | -3.9300 | 0.0000 | -0.0930 | -0.0311 |
| HTN Medication vs. rest | -0.0452 | 0.0141 | -3.2000 | 0.0010 | -0.0729 | -0.0175 |
| Limitation vs. rest | -0.2515 | 0.0126 | -19.9600 | 0.0000 | -0.2762 | -0.2268 |
| Other CV disease vs. rest | -0.0899 | 0.0150 | -6.0100 | 0.0000 | -0.1193 | -0.0606 |
| Osteoarticular pathology vs. rest | -0.1410 | 0.0181 | -7.7700 | 0.0000 | -0.1765 | -0.1054 |
| Chronic respiratory disease vs. rest | -0.0858 | 0.0070 | -12.2000 | 0.0000 | -0.0996 | -0.0720 |
| Mental disorder vs. rest | -0.0785 | 0.0085 | -9.2100 | 0.0000 | -0.0952 | -0.0618 |
| Current anxiety/depression vs. rest | -0.0950 | 0.0139 | -6.8400 | 0.0000 | -0.1222 | -0.0678 |
| Polypharmacy vs. rest | -0.0539 | 0.0282 | -1.9100 | 0.0560 | -0.1091 | 0.0014 |
| Current smoker vs. rest | -0.0271 | 0.0055 | -4.8800 | 0.0000 | -0.0379 | -0.0162 |
| Physical activity vs. rest | 0.0594 | 0.0076 | 7.7800 | 0.0000 | 0.0444 | 0.0744 |
| Normal weight vs. rest | 0.0293 | 0.0036 | 8.1400 | 0.0000 | 0.0222 | 0.0363 |
| High social class vs. rest | 0.0438 | 0.0047 | 9.2700 | 0.0000 | 0.0346 | 0.0531 |
|  |  |  |  |  |  |  |
| **predict(pr outcome(2))** | dy/dx | SE | Z | *p*>z | ILCI95% | SLCI95% |
| Year | -0.0047 | 0.0022 | -2.1600 | 0.0310 | -0.0089 | -0.0004 |
| Women vs. men | -0.0060 | 0.0054 | -1.1000 | 0.2720 | -0.0166 | 0.0047 |
| Age (years) | 0.0016 | 0.0003 | 6.2400 | 0.0000 | 0.0011 | 0.0021 |
| Married vs. rest | 0.0022 | 0.0007 | 2.9400 | 0.0030 | 0.0007 | 0.0036 |
| HTN vs. rest | 0.0265 | 0.0187 | 1.4200 | 0.1570 | -0.0102 | 0.0633 |
| A HTN H Medication vs. rest | 0.0334 | 0.0171 | 1.9500 | 0.0510 | -0.0001 | 0.0670 |
| Limitation vs. rest | 0.0165 | 0.0141 | 1.1700 | 0.2420 | -0.0111 | 0.0441 |
| Other CV disease vs. rest | 0.0226 | 0.0167 | 1.3500 | 0.1770 | -0.0102 | 0.0554 |
| Osteoarticular pathology vs. rest | 0.0455 | 0.0161 | 2.8300 | 0.0050 | 0.0139 | 0.0770 |
| Chronic respiratory disease vs. rest | 0.0079 | 0.0072 | 1.0900 | 0.2750 | -0.0063 | 0.0221 |
| Chronic mental disorder vs. rest | 0.0173 | 0.0027 | 6.4300 | 0.0000 | 0.0120 | 0.0226 |
| Current anxiety/depression vs. rest | 0.0001 | 0.0141 | 0.0100 | 0.9920 | -0.0276 | 0.0279 |
| Polypharmacy vs. rest | -0.0336 | 0.0247 | -1.3600 | 0.1730 | -0.0820 | 0.0147 |
| Current smoker vs. rest | 0.0198 | 0.0071 | 2.7800 | 0.0050 | 0.0059 | 0.0337 |
| Physical activity vs. rest | -0.0131 | 0.0014 | -9.2100 | 0.0000 | -0.0159 | -0.0103 |
| Normal weight vs. rest | -0.0113 | 0.0033 | -3.4100 | 0.0010 | -0.0178 | -0.0048 |
| High social class vs. rest | 0.0077 | 0.0042 | 1.8300 | 0.0670 | -0.0005 | 0.0159 |
|  |  |  |  |  |  |  |
|  |  |  |  |  |  |  |
| **predict(pr outcome(3))** | dy/dx | SE | Z | *p*>z | ILCI95% | SLCI95% |
| Year | -0.0096 | 0.0040 | -2.3800 | 0.0170 | -0.0175 | -0.0017 |
| Women vs. men | 0.0151 | 0.0037 | 4.1100 | 0.0000 | 0.0079 | 0.0223 |
| Age (years) | 0.0013 | 0.0002 | 7.3800 | 0.0000 | 0.0010 | 0.0017 |
| Married vs. rest | 0.0044 | 0.0014 | 3.2400 | 0.0010 | 0.0017 | 0.0071 |
| HTN vs. rest | 0.0303 | 0.0099 | 3.0600 | 0.0020 | 0.0109 | 0.0497 |
| HTN Medication vs. rest | 0.0148 | 0.0091 | 1.6200 | 0.1050 | -0.0031 | 0.0327 |
| Limitation vs. rest | 0.1125 | 0.0039 | 28.7800 | 0.0000 | 0.1048 | 0.1201 |
| Other CV disease vs. rest | 0.0471 | 0.0059 | 7.9500 | 0.0000 | 0.0355 | 0.0587 |
| Osteoarticular pathology vs. rest | 0.0733 | 0.0042 | 17.3200 | 0.0000 | 0.0650 | 0.0816 |
| Chronic respiratory disease vs. rest | 0.0597 | 0.0026 | 22.9300 | 0.0000 | 0.0546 | 0.0648 |
| Mental disorder vs. rest | 0.0355 | 0.0041 | 8.6000 | 0.0000 | 0.0274 | 0.0436 |
| Current anxiety/depression vs. rest | 0.0700 | 0.0053 | 13.2900 | 0.0000 | 0.0597 | 0.0803 |
| Polypharmacy vs. rest | 0.0604 | 0.0037 | 16.1600 | 0.0000 | 0.0531 | 0.0677 |
| Current smoker vs. rest | 0.0004 | 0.0054 | 0.0700 | 0.9450 | -0.0102 | 0.0109 |
| Physical activity vs. rest | -0.0269 | 0.0042 | -6.4700 | 0.0000 | -0.0350 | -0.0187 |
| Normal weight vs. rest | -0.0174 | 0.0029 | -6.1000 | 0.0000 | -0.0230 | -0.0118 |
| High social class vs. rest | -0.0369 | 0.0030 | -12.4000 | 0.0000 | -0.0427 | -0.0311 |
|  |  |  |  |  |  |  |
| **predict(pr outcome(4))** | dy/dx | SE | Z | *p*>z | ILCI95% | SLCI95% |
| Year | -0.0049 | 0.0022 | -2.2400 | 0.0250 | -0.0091 | -0.0006 |
| Women vs. men | -0.0004 | 0.0016 | -0.2300 | 0.8160 | -0.0036 | 0.0028 |
| Age (years) | 0.0007 | 0.0001 | 5.2800 | 0.0000 | 0.0004 | 0.0009 |
| Married vs. rest | 0.0023 | 0.0007 | 3.1200 | 0.0020 | 0.0008 | 0.0037 |
| HTN vs. rest | 0.0057 | 0.0028 | 2.0200 | 0.0440 | 0.0002 | 0.0112 |
| HTN Medication vs. rest | -0.0050 | 0.0020 | -2.5200 | 0.0120 | -0.0090 | -0.0011 |
| Limitation vs. rest | 0.0772 | 0.0030 | 26.0500 | 0.0000 | 0.0714 | 0.0830 |
| Other CV disease vs rest | 0.0135 | 0.0021 | 6.3100 | 0.0000 | 0.0093 | 0.0177 |
| Osteoarticular pathology vs. rest | 0.0187 | 0.0022 | 8.6000 | 0.0000 | 0.0144 | 0.0230 |
| Chronic respiratory disease vs. rest | 0.0148 | 0.0024 | 6.3100 | 0.0000 | 0.0102 | 0.0195 |
| Mental disorder vs. rest | 0.0181 | 0.0020 | 8.9800 | 0.0000 | 0.0141 | 0.0220 |
| Current anxiety/depression vs. rest | 0.0183 | 0.0027 | 6.7800 | 0.0000 | 0.0130 | 0.0236 |
| Polypharmacy vs. rest | 0.0207 | 0.0026 | 7.8900 | 0.0000 | 0.0156 | 0.0259 |
| Current smoker vs. rest | 0.0028 | 0.0039 | 0.7200 | 0.4730 | -0.0048 | 0.0104 |
| Physical activity vs. rest | -0.0137 | 0.0021 | -6.4800 | 0.0000 | -0.0178 | -0.0096 |
| Normal weight vs. rest | -0.0022 | 0.0021 | -1.0400 | 0.2980 | -0.0064 | 0.0019 |
| High social class vs. rest | -0.0142 | 0.0023 | -6.2700 | 0.0000 | -0.0187 | -0.0098 |

| **predict(pr outcome(5))** | dy/dx | SE | Z | *p*>z | ILCI95% | SLCI95% |
| --- | --- | --- | --- | --- | --- | --- |
| Year | -0.0020 | 0.0010 | -2.1200 | 0.0340 | -0.0039 | -0.0001 |
| Women vs. men | 0.0012 | 0.0008 | 1.5700 | 0.1180 | -0.0003 | 0.0028 |
| Age (years) | 0.0002 | 0.0001 | 2.6300 | 0.0090 | 0.0000 | 0.0003 |
| Married vs. rest | 0.0009 | 0.0004 | 2.6400 | 0.0080 | 0.0002 | 0.0016 |
| HTN vs. rest | -0.0005 | 0.0011 | -0.4400 | 0.6600 | -0.0026 | 0.0017 |
| HTN Medication vs. rest | 0.0021 | 0.0015 | 1.3800 | 0.1680 | -0.0009 | 0.0050 |
| Limitation vs. rest | 0.0453 | 0.0053 | 8.5500 | 0.0000 | 0.0349 | 0.0557 |
| Other CV disease vs rest | 0.0067 | 0.0010 | 6.3900 | 0.0000 | 0.0046 | 0.0088 |
| Osteoarticular pathology vs. rest | 0.0034 | 0.0012 | 2.7700 | 0.0060 | 0.0010 | 0.0059 |
| Chronic respiratory disease vs. rest | 0.0033 | 0.0009 | 3.7000 | 0.0000 | 0.0016 | 0.0051 |
| Mental disorder vs. rest | 0.0076 | 0.0010 | 7.5500 | 0.0000 | 0.0056 | 0.0095 |
| Current anxiety/depression vs. rest | 0.0065 | 0.0026 | 2.5600 | 0.0110 | 0.0015 | 0.0115 |
| Polypharmacy vs. rest | 0.0064 | 0.0013 | 5.0800 | 0.0000 | 0.0039 | 0.0088 |
| Current smoker vs. rest | 0.0041 | 0.0018 | 2.2800 | 0.0220 | 0.0006 | 0.0076 |
| Physical activity vs. rest | -0.0057 | 0.0008 | -7.3000 | 0.0000 | -0.0073 | -0.0042 |
| Normal weight vs. rest | 0.0016 | 0.0012 | 1.4300 | 0.1540 | -0.0006 | 0.0039 |
| High social class vs. rest | -0.0004 | 0.0013 | -0.3000 | 0.7640 | -0.0030 | 0.0022 |

HTN: arterial hypertension; CV: cardiovascular; SE: Standard Error; IL/SL CI95%. Inferior/Superior limit of the 95% confidence interval

outcome(1)= Perception of health status “very good”.

outcome(2)= Perception of health status “ good”.

outcome(3)= Perception of health status “fair”.

outcome(4)= Perception of health status “bad”.

outcome(5)= Perception of health status “very bad”.
