# Supplementary material for: Association between hypertension and self-perception of health status: Findings from a decade population-based survey in Spanish adults
Source: PLoS One. 2025 May 7;20(5):e0322577. doi: 10.1371/journal.pone.0322577 (PMC12057956; doi:10.1371/journal.pone.0322577)
Supplement: Attachment — Submitted filename: HTN and health perception_PLOS_REVIEWED 30JUL2024.docx [file pone.0322577.s005.docx]

**Does a diagnosis of arterial hypertension affect the perception of health status? A one-decade-long study in the Spanish population.**

**Authors:**

Jesus Martín-Fernández* ^1,2,3,4^, Tamara Alonso-Safont ^5,6^, Elena Polentinos-Castro^2,3,4,7^, Gemma Rodríguez-Martínez ^8^, Mª Isabel González Anglada^2,9^, Amaia Bilbao-González, Isabel del-Cura-González^2,3,4,7^ .

1. Oeste Family and Community Care Teaching Unit, Primary Care Assistance Management, Madrid Health Service, Madrid, Spain
2. Department of Medical Specialties and Public Health, Faculty of Health Sciences, Rey Juan Carlos University, Madrid, Spain
3. Research Network on Chronicity, Primary Care and Health Promotion-RICAPPS (RICORS). ISCIII, Spain
4. Gregorio Marañón Health Research Institute (IISGM), Madrid, Spain
5. Technical Directorate of Health Information Systems, Primary Care Management, Madrid Health Service, Madrid, Spain
6. Doctoral student, Doctoral program in Health Sciences, Rey Juan Carlos University, Madrid, Spain
7. Research Unit, Primary Care Assistance Management. Madrid Health Service, Madrid, Spain
8. Infante Don Luis Health Center, Primary Care Assistance Management, Madrid Health Service, Madrid, Spain
9. Internal Medicine Service. Alcorcón Foundation University Hospital, Madrid
10. Osakidetza Basque Health Service, Basurto University Hospital, Research and Innovation Unit, Bilbao, Spain
11. Kronikgune Health Services Research Institute, Barakaldo, Spain
12. Department of Medicine, Faculty of Health Sciences, University of Deusto, Bilbao, Spain

*Corresponding author:

Email: [jmfernandez@salud.madrid.org](mailto:jmfernandez@salud.madrid.org)

**Abstract**

Objective:

This study, conducted in the community setting, aimed to assess and discuss the association of being diagnosed with arterial hypertension with self-perceived health status and potential explanative factors.

Methods:

Cross-sectional observational study using the 2011-2012 and 2017 Spanish National Health Surveys and the 2020 European Health Interview Survey for Spain as data sources. Health perception was categorised as very good, good, fair, bad, or very bad. The independent variables recorded demographic, social, clinical, and lifestyle information. The associations between variables were evaluated via a generalisation of an ordered logit model.

Results:

A total of 66,168 subjects were included (21,007 in 2011, 23,089 in 2017, and 22,072 in 2020), 26.74% of whom were diagnosed with hypertension, 53.7% were women, and the average age was 53.23 (19.03) years. Around one in five people in the general population reported a "very good" health status.. The probability of reporting a “very good” health condition decreased with a diagnosis of hypertension (5.4%; CI95%: 3.2–7.6%) and hypertensive medication (3.1%; CI95%: 1.0–5.2). Such associations were not related to age, gender, social group, other chronic conditions or limitations, or various lifestyle habits. In contrast, no association was found with reporting a “bad” or “very bad” health status.

Conclusion:

Being diagnosed with hypertension and prescription of antihypertensive medication are associated with a lower probability of reporting a “very good” health status, irrespective of other comorbidities or complications related to the diagnosis.

Keywords: hypertension; health status; health-related quality of life; population health

**Introduction**

Arterial hypertension (HTN) is one of the most prevalent conditions in adults, reaching 32% of women and 34% of men over 30 years of age (1). This entails a significant disease burden, making HTN responsible for the loss of 143 million disability-adjusted life years globally in 2015, an increase of >30% versus those in 1990 (2). This growing prevalence of HTN is partly explained by the increase in obesity and unhealthy lifestyle habits (3,4) as well as the population ageing, as subjects who reach the average life expectancy without HTN are >90% more likely to develop the disease throughout their remaining life (5).

Numerous studies have demonstrated the association of HTN with various conditions, especially cardiovascular diseases (2,6,7) that lower life expectancy (8–11). HTN is generally asymptomatic in the absence of comorbidities. This can lead patients to erroneously believe that the disease is benign, which entails implications when adopting permanent lifestyle (12) or pharmacological (13–15) recommendations.

Various constructs have been designed to understand the repercussions that a health situation or lack thereof has on the patient’s life. Health-related quality of life (HRQoL) is one such construct, which can be defined as the self-perceived health condition and how disease or treatments affect the well-being and functionality of patients (16). The concept of HRQoL overlaps with the self-perception of health status. Therefore, HRQoL could be defined as the way in which health affects quality of life or used to state a preference associated with a certain health status (17).

Studies have shown a negative impact of HTN on HRQoL (18–21), although this is believed to be caused by the awareness of the consequences of suffering the disease and not by the diagnosis itself (20). Other research has reported that neither the diagnosis nor the stage of the disease affect the perception of health, in either its physical or mental dimensions, which is attributed to the absence of symptoms, at least in patients with uncomplicated hypertension (22). The mentioned association has also been related to the need to take medication chronically (23).

The perception of health status changes throughout life (24). Therefore, such perception should be affected by HTN, as it becomes more prevalent with age. However, a "recalibration" phenomenon has been shown in hypertensive patients, in which a lower functional status is assumed as "normal" with ageing (25). Therefore, the perceived health in older adults with HTN appears to be the result of a realistic appraisal of age, chronic diseases, recent acute events, functionality, and stress (26).

Whether the self-perception of health worsens in hypertensive patients is still unclear, and so is whether this alteration is due to the diagnosis, chronic medication, or comorbidities. The aim of this study conducted in the community setting was to analyse the association of HTN with a worse perception of health and determine the potential explanative factors.

**Methods**

Design

An observational study was designed. The 2011-2012 and 2017 Spanish National Health Surveys (ENSE) and the 2020 European Health Interview Survey (EHIS) for Spain were used as secondary sources. , as their data is anonymised and publically available (<https://www.ine.es/dyngs/INEbase/es/categoria.htm?c=Estadistica_P&cid=1254735971047>).

A stratified sampling was performed in three phases: first, selection of census sections (2,000 subjects in 2011 and 2,500 participants in 2017 and 2020); second, selection of 12 households from the 2011 section and 15 from the 2017 and 2020 census sections; third, from each household, an adult >15 years old was selected and a minor, if present (27–29). Only the information on adults was collected from the surveys.

Variables

The outcome variables were collected through equally formulated questions from the three surveys. The dependent variable was self-perceived health status, which was categorised into five levels: very good, good, fair, bad, and very bad. The independent variables were the hypertension diagnosis together with demographic, social, clinical, and lifestyle data.

Age, gender, and residence region were recorded as demographic variables. The social data included nationality (Spanish/foreigner), marital status (single, married, widow/separated/divorced), and social class in six levels based on the professional category (30) (with levels I and II considered “high class”).

Lifestyle-related variables were also collected, such as smoking habit (current versus former smoker/never smoked), physical activity (regular if performed several times a month), and body mass index (BMI) (underweight, normal weight, overweight, and obese, comparing normal weight versus the rest).

The recorded clinical variables were the existence of a HTN diagnosis, prescription for HTN, other cardiovascular pathologies (myocardial infarction, heart pathologies, or cerebrovascular accidents), osteoarticular pathologies (arthrosis, arthritis, rheumatism, or chronic back pain), chronic mental disorder (depression, anxiety, or other conditions), and recent diagnosis of anxiety or depression (last 12 months).

The subject's perception of limitation due to illness (severe, mild, and no limitation, comparing the latter versus the first two) was also included. The number of prescribed chronic drugs other than antihypertensive medication was obtained and polypharmacy was considered present for ≥5 different types of treatment.

Analysis

The variable distributions were described by absolute values and frequencies in the case of qualitative ones and by measures of central tendency and dispersion in the case of continuous ones.

The self-reported perception of health status was analysed via a five-category variable: very good (Y=1), good (Y=2), fair (Y=3), bad (Y=4), and very bad (Y=5). As these are ordered according to the level of health status (inversely), a standard ordered Logit model was selected. The probability that Y is included in a category is calculated as (31):

P (Y=1) = 1 - g (Xβ_1_)

P (Y=m) = g ( Xβ_m-1_) - g( Xβ_m_), m=2,…,J-1

P (Y=J) = g (Xβ_J-1_)

where X represents the matrix of covariates and β the vector of coefficients. The term Y represents the discrete categorisation of a non-observable continuous latent variable Y*, which stands for the real perception of health status and adopts the following structural shape: Y* = Xβ + ε. The proportional odds assumption, namely the strength of the association is equal for each category, must be proven to validate this model. After adjusting the standard ordered Logit model, the Brant test failed to verify this assumption (32), therefore showing the inadequate use of this model.

Subsequently, a partial proportional odds model (33), generalisation of the ordered Logit model, was formulated, which relaxes the proportional odds model for a subset of independent variables through different categories of the outcome variable. Its best fit was tested using the Akaike Information Criteria, Bayes Information Criteria, and pseudo-adjusted R^2^, following the suggestions by Kass and Raftery (34). Standard errors were estimated using robust methods to account for potential heteroscedasticity (35). Although this model can be characterised through its coefficients, it can also be interpreted as a nonlinear probability model that allows examining the determinants and the likelihood of occurrence of each outcome, the marginal effects, and particular cases (36). Therefore, the predictions for certain subgroups of subjects and the marginal effects can be presented, that is, the variability in the probability of expressing a specific category of health status with a change of one unit in the independent variable, assuming the rest of covariates at their mean values. All analyses were performed with the Stata 14.2® software.

Ethical and legal aspects

This study followed the Good Practice Guidelines for clinical research. Consent to collect data was obtained from interviewed people during the creation of the original data sources by the National Institute of Statistics and the Health Ministry (27–29). The authors never had access to the personal data of the original respondents and accessed the anonymised information during April 2023. The Ethics Committee for Research from Hospital Universitario Fundación Alcorcón approved the study (ref. 23/62) and the procedures related to data collection.

**Results**

A total of 66,168 subjects were included: 21,007 in 2011, 23,089 in 2017, and 22,072 in 2020. The average age was 53.23 (SD 19.03, median 53, interquartile range 39–68) years and 53.7% were women. A diagnosis of HTN was found in 17,692 subjects (5,391 in 2011, 6,244 in 2017, and 6,057 in 2020), which amounted to 26.74% of the sample. Of those subjects not diagnosed with HTN, 24.10% and 52.44 % reported a “very good” and “good” perception of their health status, respectively, versus 5.53% and 40.18% of people with HTN. Table 1 shows the characteristics of the overall sample.

Table 1. Characteristics of the study sample.

| **Survey year** | **2011**  **N=21,007** | **2017**  **N=23,089** | **2020**  **N=22,072** | **Total**  **N=66,168** |
| --- | --- | --- | --- | --- |
| Women | 11,358 (54.07%) | 12,494 (54.11%) | 11,682 (52.93%) | 35,534 (53.70%) |
| Average age (SD) | 51.60 (19.09) | 53.44 (18.89) | 54.57 (19.01) | 53.23 (19.03) |
| Nationality  (Spanish) | 19,682 (93.69%) | 21,642 (93.73%) | 20,758 (94.05%) | 62,082 (93.82%) |
| Marital status  (married) | 10,979 (52.26%) | 12,465 (53.99%) | 11,301 (51.20%) | 34,745 (52.51%) |
| Social class (high) | 3,712 (17.67%) | 4,053 (17.55%) | 3,980 (18.03%) | 11,745 (17.75%) |
| Health status |  |  |  |  |
| Very good | 3,736 (17.78%) | 4,190 (18.15%) | 4,736 (21.46%) | 12,662 (19.14%) |
| Good | 10,532 (50.14%) | 11,145 (48.27%) | 10,851 (49.16%) | 32,528 (49.16%) |
| Fair | 4,768 (22.70%)) | 5,531 (23.96%) | 4,593 (20.81%) | 14,892 (22.51%) |
| Bad | 1,574 (7.49%) | 1,724 (7.47%) | 1,493 (6.76%) | 4,791 (7.24%) |
| Very bad | 397 (1.89%) | 499 (2.16%) | 399 (1.81%) | 1295 (1.96%) |
| Smoker |  |  |  |  |
| Yes | 5,316 (25.33%) | 5,398 (23.40%) | 4,638 (21.05%) | 15,352 (23.23%) |
| No | 15668 (74,67%) | 17,669 (76,60%) | 17,396 (78.95%)) | 50,733 (76.77%)) |
| Regular physical activity |  |  |  |  |
| No | 16,975 (80.86%) | 17,920 (77.65%) | 16,590 (75.24%) | 51,485 (77.88%) |
| Yes | 4,016 (19.14%) | 5,169 (22.35%) | 5,460 (24.76%) | 14,635 (22.12%) |
| Weight |  |  |  |  |
| Underweight | 375 (1.97%) | 475 (2.16%) | 407 (1.95%) | 1,257 (2.03%) |
| Normal weight | 8,176 (42.88%) | 9,301 (42.24%) | 8,964 (42.88%) | 26,441 (42.65%) |
| Overweight | 7,171 (37.61%) | 8,333 (37.84%) | 8,156 (39.01%) | 23,660 (38.16%) |
| Obesity | 3,347 (17.55%) | 3,910 (17.76%) | 3,379 (16.16%) | 10,636 (17.16%) |
| Limitation due to illness |  |  |  |  |
| No | 16,288 (77.56%) | 16,308 (70.64%) | 15,955 (72.32%) | 48,551 (73.40%) |
| Not severe | 3,822 (18.20%) | 5,476 (23.72%) | 4,835 (21.92%) | 14,133 (21.37%) |
| Severe | 890 (4.24%) | 1,301 (5.64%) | 1,271 (5.76%) | 3,462 (5.23%) |
|  |  |  |  |  |
| HTN diagnosis | 5,391 (25.66%) | 6,244 (27.04%) | 6,057 (27.44%) | 17,692 (26.74%) |
| Other cardiovascular disease | 2,070 (9.85%) | 2,588 (11.21%) | 2,357 (10.68%) | 7,015 (10.60%) |
| Respiratory disease | 1,922 (9.15%) | 2,175 (9.42%) | 1,820 (8.25%) | 5,917 (8.94%) |
| Osteoarticular disease | 5,850 (27.85%) | 6,208 (26.89%) | 4,785 (21.68%) | 16,843 (25.45%) |
| Mental disorder | 2,844 (13.54%) | 3,551 (15.38%) | 2,888 (13.08%) | 9,283 (14.03%) |
| Current diagnosis of anxiety/depression | 2,381 (11.33%) | 2,807 (12.16%) | 2,311 (10.47%) | 7,499 (11.33%) |
| Medication for HTN | 4,398 (20.94%) | 5,775 (25.01%) | 5,380 (24.37%) | 15,553 (23.51%) |
| Polypharmacy | 1,772 (8.44%) | 2,214 (9.59%) | 1,811 (8.20%) | 5,797 (8.76%) |

SD: standard deviation; HTN: arterial hypertension.

The final model was constructed incorporating all the variables in blocks except nationality, which was not associated with the outcome. The final model included 61,933 cases, adjusted for the complete year of the survey (pseudo-R^2^ 0.2306) (details can be found in Annex 1). This output can be interpreted as a succession of independent regressions where the coefficients express the association with a worse outcome. First, outcome 1 (very good) is compared versus the remaining possibilities; subsequently, outcomes 1 and 2 (very good and good) are compared against the rest; and so forth. The association between being diagnosed with HTN and the self-perceived health status did not fit the proportional odds model and its association was therefore calculated for each stratum. This association, despite being negative for all the strata, was stronger for the best health status. Something analogous occurred with the association between antihypertensive medication and perception of health status. Annex 1 shows which variables violated the proportional odds assumption and which did not in the final model.

Table 2 shows the predicted likelihood of reporting a specific health status for the complete sample and particular subgroups, adjusted for the characteristics of each subject. The probability of expressing a “very good” health status was only 12.8% in hypertensive patients with pharmacological treatment versus 15.0% in hypertensive subjects globally and 20.1% in non-hypertensive subjects. The adjusted likelihood of expressing a “poor” or “very poor” health status did not significantly differ between hypertensive subjects, with or without medication prescription, and the general population.

Table 2. Mean probability of reporting a specific health status in non-hypertensive and hypertensive patients, both with and without related medication (Annex 1 model).

| **Health status** | **Very good**  **%**  **(CI95%)** | **Good**  **%**  **(CI95%)** | **Fair**  **%**  **(CI95%)** | **Bad**  **%**  **(CI95%)** | **Very bad**  **%**  **(CI95%)** |
| --- | --- | --- | --- | --- | --- |
| Overall sample | 19.49  (19.37– 19.62) | 50.01  (49.85– 50.16) | 21.91  (21.76– 22.05) | 6.77  (6.68– 6.86) | 1.82  (1.79– 1.86) |
|  |  |  |  |  |  |
| No hypertension | 20.13%  (18.34– 21.92%) | 50.44%  (48.85– 52.02) | 21.01%  (20.24– 21.78) | 6.63%  (6.14– 7.11) | 1.8%  (1.44– 2.16) |
|  |  |  |  |  |  |
| Hypertension with medication | 12.75  (11.04– 14.46) | 53.35  (51.61– 55.09) | 25.35  (24.11– 26.59) | 6.69  (6.18– 7.19) | 1.87  (1.54– 2.2) |
|  |  |  |  |  |  |
| Hypertension | 15.03  (12.6– 17.46) | 51.92  (49.41– 54.42) | 24.25  (22.76– 25.75) | 6.95  (6.37– 7.53) | 1.85  (1.54– 2.16) |
|  |  |  |  |  |  |

CI95%: Confidence interval at 95%

Table 3 displays the percentage differences in the likelihood of reporting a certain health status for different conditions other than HTN. These probabilities stem from the model in Appendix 1 and are adjusted for the rest of the variables at their mean values. A diagnosis of HTN was associated with a decrease of 5.4 percentage points in the probability of expressing "very good" health while no association was found with "bad" or "very bad" health reports. Taking medication for HTN was also associated with a decrease of 3.11 points in the probability of expressing a "very good" health status.

Suffering a chronic osteoarticular disease was the clinical condition most strongly associated with a lower probability of reporting a “very good” health status (decrease of 12.12%), followed by cardiovascular disease or being diagnosed with anxiety or depression over the last year (decrease of >7.5%).

Table 3. Variability in the probability of reporting a specific perception of health status for different clinical conditions, adjusted by the remaining characteristics (Table 1 model).

| **Health status** | **Very good**  **%**  **(CI95%)** | **Good**  **%**  **(CI95%)** | **Fair**  **%**  **(CI95%)** | **Bad**  **%**  **(CI95%)** | **Very bad**  **%**  **(CI95%)** |
| --- | --- | --- | --- | --- | --- |
| Hypertension | -5.4  (-7.64– -3.15) | 1.90*  (-0.40– 4.20) | 3.12  (1.68– 4.56) | 0.33*  (-0.29– 0.94) | 0.05*  (-0.22– 0.32) |
| Cardiovascular disease | -7.84  (-9.78– -5.89) | 0.66*  (-1.63– 2.96) | 5.03  (3.92– 6.15) | 1.37  (1– 1.74) | 0.77  (0.54– 0.99) |
| Chronic osteoarticular  disease | -12.15%  (-15.28– -9.0) | 2.25%*  (-0.41– 4.91) | 7.27%*  (6.42– 8.11) | 2.18%  (1.78– 2.59) | 0.45%  (0.17– 0.73) |
| Chronic respiratory  disease | -6.86  (-8.27– -5.45%) | -0.99*  (-2.36– 0.38%) | 5.52  (4.74– 6.3%) | 1.85  (1.39– 2.3%) | 0.49  (0.28– 0.69%) |
| Chronic mental  disorder | -6.5  (-7.72– -5.28%) | 0.37*  (-0.07– 0.82%) | 3.31  (2.68– 3.95%) | 2  (1.65– 2.34%) | 0.82  (0.61– 1.03%) |
| Anxiety/depression diagnosis in the last year | -7.67  (-9.8– -5.54) | -1.66 *  (-3.59– 0.27) | 6.71  (5.66– 7.75) | 2.02  (1.61– 2.44) | 0.6  (0.25– 0.95) |
| Polypharmacy | -7.62  (-13.61– -1.6%) | -1.89*  (-7.18– 3.41%) | 6.02  (5.07– 6.97%) | 2.56  (1.93– 3.19%) | 0.92  (0.72– 1.12%) |
| Antihypertensive drugs prescription | -3.11  (-5.2– -1.02) | 2.08*  (-0.01– 4.18) | 1.44  (0.02– 2.87) | -0.46*  (-1.07– 0.14) | 0.04*  (-0.23– 0.32) |

CI95%: Confidence interval at 95%

*: do not reach statistical significance set at *p*<0.05.

**Discussion**

The diagnosis of HTN is associated with a worse self-perception of health status in the general Spanish population. This association reaches significance when reporting the best health status ("very good"), but not with perceptions of "very bad" health status. The medication for HTN shows similar associations but of lesser magnitude, independently of the disease diagnosis. The observed associations were adjusted for those characteristics that were found to modify the perception of health status, such as age, gender, social group, concomitant chronic diseases or limitations, and lifestyles (24,37).

Former studies reported the negative association between HTN and the perception of health status, although without isolating the potential influence of comorbidity or other variables (18,19). These studies did show that chronic disease was associated with a worse perception of health status in hypertensive patients, but not whether it was differentially so with respect to the general population. The described association has been established in other larger studies. However, whether the decrease in the perceived health status is due to the diagnosis itself or to concern about the disease has not been determined, nor the role of medication (20). In Spain, a study conducted more than 15 years ago found no association between the diagnosis of HTN and a worse perception of HRQoL nor with the prescription of medication, except in a subgroup of women who expressed concern about the diagnosis (38). The findings of the present study, which differed from that research, also allow estimating the magnitude of the association comparatively. The diagnosis of HTN decreases the probability of expressing a "very good" health status by 5.4 percentage points, a decrease that reaches >12 and >7.5 points in the cases of chronic osteoarticular disease or recent diagnosis of anxiety or depression, respectively. The very strong association between osteoarticular pathologies and the perception of HRQoL has been previously documented in European countries (39) and Spain (40), similarly to diagnoses of anxiety or depression (41,42). Therefore, the impact of the diagnosis of HTN on the perception of the best health status could be comparatively considered significant. In contrast, this diagnosis was not associated with a greater probability of reporting a “bad” or “very bad” health status, as is the case for the other mentioned pathologies.

The intake of medication for HTN in the absence of comorbidities was associated with a decrease of 3.11 percentage points in the probability of expressing a "very good" health status. This is approximately equivalent to 40% of the magnitude of the inverse association between polypharmacy and self-reported HRQoL (43). However, contrary to polypharmacy, the intake of medication for HTN was not associated with the perception of a "bad" or "very bad" health status.

Both the diagnosis of HTN and the prescribed medication appear to be independently associated with a less positive health perception. This study did not assess whether better control of the disease improves this perception, or whether this association is affected by differences in coping with the disease, as proposed by other authors (14,15). The present findings point to confirm that the referred associations were not solely related to the existence of comorbidity or ageing and that the perception of health in hypertensive patients worsens even if their general physical condition is not affected (19). Whether the worse perception of health status is due to concern about the disease, blood pressure values, or the recommended lifestyle changes could not be established. However, a conclusion could be reached that this impact was not due to disease-related complications and that its magnitude was moderate compared to other conditions, as shown in other research (20).

The implications of these outcomes are clear. The approach of health professionals to hypertensive patients, especially in the community setting, should consider that this diagnosis worsens the perception of health. Therefore, interventions that improve coping with the disease can be positive. The findings are also of interest when planning health provision, which should consider the patient’s perspective.

Among the study limitations is its cross-sectional design, which allows assessing associations but not causality. All the data was self-reported and were therefore subject to various reporting biases. In addition, the lack of data on blood pressure figures prevented classifying the diagnosis into different degrees. As a strength, the data was collected over a lengthy period and from the most representative sample of the Spanish population. However, an underrepresentation of socially excluded groups could be present due to the methodology of the surveys, which are carried out at the subjects’ homes.

In conclusion, this research established the association between the diagnosis of HTN and a lower probability of expressing perceptions of "very good" health status at the population level. Such association was not mediated by the presence of other comorbidities or related complications. Medication for HTN was also found to be independently associated with expressions of less satisfactory health status.

**References**

1.	Zhou B, Carrillo-Larco RM, Danaei G, Riley LM, Paciorek CJ, Stevens GA, et al. Worldwide trends in hypertension prevalence and progress in treatment and control from 1990 to 2019: a pooled analysis of 1201 population-representative studies with 104 million participants. Lancet. 2021;398(10304):957–80.

2.	Forouzanfar MH, Liu P, Roth GA, Ng M, Biryukov S, Marczak L, et al. Global burden of hypertension and systolic blood pressure of at least 110 to 115mmHg, 1990-2015. JAMA. 2017;317(2):165–82.

3.	Kringeland E, Tell GS, Midtbø H, Haugsgjerd TR, Igland J, Gerdts E. Factors associated with increase in blood pressure and incident hypertension in early midlife: the Hordaland Health Study. Blood Press. 2020;29(5):267–75.

4.	Shihab HM, Meoni LA, Chu AY, Wang N-YNY, Ford DE, Liang KYK-Y, et al. Body Mass Index and Risk of Incident Hypertension Over the Life Course: The Johns Hopkins Precursors Study. Circulation. 2012 Dec 18;126(25):2983–9.

5.	O’Brien E. The Lancet Commission on hypertension: Addressing the global burden of raised blood pressure on current and future generations. J Clin Hypertens. 2017;19(6):564–8.

6.	Shah NS, Molsberry R, Rana JS, Sidney S, Capewell S, O’Flaherty M, et al. Heterogeneous trends in burden of heart disease mortality by subtypes in the United States, 1999-2018: observational analysis of vital statistics. BMJ. 2020 Aug 13;370:m2688.

7.	Vaughan AS, Coronado F, Casper M, Loustalot F, Wright JS. County‐Level Trends in Hypertension‐Related Cardiovascular Disease Mortality—United States, 2000 to 2019. J Am Heart Assoc. 2022 Apr 5;11(7).

8.	Sepanlou SG, Sharafkhah M, Poustchi H, Malekzadeh MM, Etemadi A, Khademi H, et al. Hypertension and mortality in the Golestan Cohort Study: A prospective study of 50000 adults in Iran. Vol. 30, J Hum Hypertens. Macmillan Publishers Limited; 2016. p. 260–7.

9.	da Silva TLN, Klein CH, Nogueira A da R, Salis LHA, de Souza E Silva NA, Bloch KV. Cardiovascular mortality among a cohort of hypertensive and normotensives in Rio de Janeiro - Brazil - 1991-2009. BMC Public Health. 2015;15:623.

10.	Lawlor DA, Kim L, Morris R, Amuzu A, Whincup P, Ebrahim S. Survival with treated and well-controlled blood pressure: Findings from a prospective cohort study. PLoS One. 2011;6(4).

11.	Park S, Han K, Lee S, Kim Y, Lee Y, Kang MW, et al. Cardiovascular or mortality risk of controlled hypertension and importance of physical activity. Heart. 2021 Sep;107(18):1472–9.

12.	Alefan Q, Huwari D, Alshogran OY, Jarrah MI. Factors affecting hypertensive patients’ compliance with healthy lifestyle. Patient Prefer Adherence. 2019;13:577–85.

13.	Ross S, Walker A, MacLeod MJ. Patient compliance in hypertension: role of illness perceptions and treatment beliefs. J Hum Hypertens. 2004 Sep 1;18(9):607–13.

14.	Alkerwi A, Pagny S, Lair ML, Delagardelle C, Beissel J. Level of Unawareness and Management of Diabetes, Hypertension, and Dyslipidemia among Adults in Luxembourg: Findings from ORISCAV-LUX Study. PLoS One. 2013;8(3):1–7.

15.	Saarti S, Hajj A, Karam L, Jabbour H, Sarkis A, El Osta N, et al. Association between adherence, treatment satisfaction and illness perception in hypertensive patients. J Hum Hypertens. 2016 May 27;30(5):341–5.

16.	Urzúa M A, Urzua A. Calidad de vida relacionada con la salud: Elementos conceptuales. Rev Med Chile. 2010 Mar;138(3):358–65.

17.	Karimi M, Brazier J. Health, Health-Related Quality of Life, and Quality of Life: What is the Difference? Pharmacoeconomics. 2016 Jul 18;34(7):645–9.

18.	Alefishat E, S. Jarab A, Abu Farha R. Factors affecting health‐related quality of life among hypertensive patients using the EQ‐5D tool. Int J Clin Pract. 2020 Sep 14;74(9):1–8.

19.	Kaliyaperumal S, Hari SB, Siddela PK, Yadala S. Assessment of quality of life in hypertensive patients. J Appl Pharm Sci. 2016;6(5):143–7.

20.	Trevisol DJ, Moreira LB, Kerkhoff A, Fuchs SC, Fuchs FD. Health-related quality of life and hypertension: A systematic review and meta-analysis of observational studies. J Hypertens. 2011;29(2):179–88.

21.	Shah R, Patel J, Shah A, Desai G, Buch J. Determinants of health-related quality of life in patients with hypertension. Natl J Physiol Pharm Pharmacol. 2020;10(4):1.

22.	Katsi V, Kallistratos MS, Kontoangelos K, Sakkas P, Souliotis K, Tsioufis C, et al. Arterial hypertension and health-related quality of life. Front Psychiatry. 2017;8(DEC):1–7.

23.	Trevisol DJ, Moreira LB, Fuchs FD, Fuchs SC. Health-related quality of life is worse in individuals with hypertension under drug treatment: results of population-based study. J Hum Hypertens. 2012 Jun 19;26(6):374–80.

24.	Martín-Fernández J, Ariza-Cardiel G, Polentinos-Castro E, Sanz-Cuesta T, Sarria-Santamera A, del Cura-González I. Explaining differences in perceived health-related quality of life: a study within the Spanish population. Gac Sanit. 2018 Sep;32(5):447–53.

25.	Chen H, Zhu L, Zhou R, Liu P, Lu X, Patrick DL, et al. Detecting response shift in health-related quality of life measurement among patients with hypertension using structural equation modeling. Health Qual Life Outcomes. 2021;19(1):1–10.

26.	Lewis LM, Riegel BJ. Determinants of perceived health in older adults with hypertension. Hear Lung J Acute Crit Care. 2010;39(1):41–9.

27.	Instituto Nacional de Estadistica. Ministerio de Sanidad Servicios Sociales e Igualdad. Metodología de la Encuesta Nacional de Salud 2011–2012. 2013. p. 1–12.

28.	Ministerio de Sanidad Consumo y Bienestar Social. Encuesta Nacional de Salud 2017 Metodología. Encuesta Nac Salud. 2017;64.

29.	Instituto Nacional de Estadística. Encuesta Europea de Salud en España 2020. Instituto Nacional de Estadistica. 2020. p. 1–6.

30.	Domingo-Salvany A, Bacigalupe A, Carrasco JM, Espelt A, Ferrando J, Borrell C. Propuestas de clase social neoweberiana y neomarxista a partir de la Clasificación Nacional de Ocupaciones 2011. Gac Sanit. 2013 May;27(3):263–72.

31.	Greene WH. Discrete choice and event counts. In: Greene WH, editor. Econometric Analysis. 7th ed. Pearson; 2012. p. 800–72.

32.	Brant R. Assessing proportionality in the proportional odds model for ordinal logistic regression. Biometrics. 1990;1171–8.

33.	Williams R. Generalized Ordered Logit/ Partial Proportional Odds Models for Ordinal Dependent Variables. Stata J. 2006;6(1):58–82.

34.	Kass RE, Raftery AE. Bayes Factors. J Am Stat Assoc. 1995 Jun;90(430):773–95.

35.	Williams RL. A Note on Robust Variance Estimation for Cluster-Correlated Data. Biometrics. 2000 Jun;56(2):645–6.

36.	Williams R. Understanding and interpreting generalized ordered logit models. J Math Sociol. 2016 Jan 2;40(1):7–20.

37.	Oliva-Moreno J, Zozaya N, López-Valcárcel BG. Opposite poles: A comparison between two Spanish regions in health-related quality of life, with implications for health policy. BMC Public Health. 2010 Dec 26;10(1):576.

38.	Banegas J, Guallar_Castillón P, Rodríguez-Artalejo F, Graciani A, López-García E, Ruilope L. Association Between Awareness, Treatment, and Control of Hypertension, and Quality of Life Among Older Adults in Spain. Am J Hypertens. 2006 Jul;19(7):686–93.

39.	Wu M, Brazier JE, Kearns B, Relton C, Smith C, Cooper CL. Examining the impact of 11 long-standing health conditions on health-related quality of life using the EQ-5D in a general population sample. Eur J Heal Econ. 2015 Mar 10;16(2):141–51.

40.	Martín-Fernández J, García -Maroto R, Bilbao A, García-Pérez L, Gutiérrez-Teira B, Molina-Siguero A, et al. Impact of lower limb osteoarthritis on health-related quality of life: A cross-sectional study to estimate the expressed loss of utility in the Spanish population. PLoS One. 2020 Jan 24;15(1):e0228398.

41.	Martín-Fernández J, Del Nido-Varo LP, Vázquez-de-la-Torre-Escalera P, Candela-Ramírez R, Ariza-Cardiel G, García-Pérez L, et al. Health Related Quality of Life in Major Depressive Disorder: evolution in time and factors associated. Actas Esp Psiquiatr. 2022 Jan;50(1):15–26.

42.	Kolovos S, Bosmans JE, van Dongen JM, van Esveld B, Magai D, van Straten A, et al. Utility scores for different health states related to depression: individual participant data analysis. Qual Life Res. 2017 Jul 4;26(7):1649–58.

43.	Aljeaidi MS, Haaksma ML, Tan ECK. Polypharmacy and trajectories of health-related quality of life in older adults: an Australian cohort study. Qual Life Res. 2022 Sep 27;31(9):2663–71.

**Declarations**

**Financial Disclosure Statement**

The translation of this manuscript study was funded by the Instituto de Salud Carlos III (ISCIII) (ref. PI1800370) and co-financed by the European Union.

The authors have received no other financial support for the elaboration of this manuscript.
