# Supplementary material for: Association between hypertension and self-perception of health status: Findings from a decade population-based survey in Spanish adults
Source: PLoS One. 2025 May 7;20(5):e0322577. doi: 10.1371/journal.pone.0322577 (PMC12057956; doi:10.1371/journal.pone.0322577)
Supplement: Attachment — Submitted filename: To the Editorial Team_final.docx [file pone.0322577.s007.docx]

To the Editorial Team:

We sincerely appreciate the thorough review of our manuscript now entitled, following the suggestion of Reviewer 1 “How a diagnosis of arterial hypertension influences health status perception: findings from a decade-long study in the Spanish population” We are sincerely grateful for the efforts of the editors and reviewers, whose work has substantially enhanced the manuscript. We are confident that these improvements will contribute significantly to the final outcome.

First of all, we have slightly modified the wording of the objective to better align it with the idea we wanted to convey. Its wording is now as follows: “This study, conducted in the community setting, aimed to assess and discuss how a diagnosis of arterial hypertension affects self-perceived health status, examining the association with potential explanatory factors and comparing its impact with that of other chronic conditions”.

Additionally, we would like to note that the authors have recognized the potential for improvement in the data analysis during the review process. The data comes from three national surveys in which sampling was conducted using clusters to ensure representativeness at the Autonomous Community level. This means that each subject has a different level of representativeness, which is accounted for in a weighting factor. Consequently, all analyses have been corrected to take this weighting factor into account. As a result, the findings in Table 1 and the model (Appendix 1, Appendix 2 and Tables 2 and 3) differ slightly from the original results, although the direction and magnitude of the reported relationships remain unchanged.

We will now proceed to address the suggestions and corrections proposed by Editor and reviewers.

**ACADEMIC EDITOR:
Both reviewers felt that the novelty of this work is rather limited.
Specifically, a number of factors, including the level of literacy or level of education of the population, and to some extent the socioeconomic status of the population, should be elaborated to highlight the impact and novelty of the study.
The results and interpretation of the model should be revised for better clarification.**

We appreciate the opportunity to highlight the novelty of this study. As we detail further (see response to the reviewers), it was not clear whether a diagnosis of hypertension (HTN) affects the perception of health status when there are no cardiovascular complications. Some studies in our country question this issue. Additionally, studies that claim this impact exists are unsure whether to attribute it to labeling effects, medication, potential side effects of the medication, or certain complications. In this work, we clarify some, though not all, of these aspects. We found that the diagnosis already affects the perception of health status, regardless of whether medication is prescribed and without being due to potential complications of hypertension.

We agree on the importance of adjusting health status perception for socioeconomic characteristics such as education level or social class. In this study, a classification of social class based on occupation was used as an adjustment variable, which includes professionals with higher education in the “higher social class” group. We explain these details and their implications in our response to the reviewers below.

Regarding the results and interpretation of the model, we have added the results in a new appendix in their most interpretable form, as marginal effects. These results show how the probability of expressing a certain category (perception of health status) changes when the explanatory variable is modified, while keeping the other variables constant at their means (we present the marginal effects at the means). It is worth noting that one of the reviewers mentioned being unable to evaluate the model due to not having access to it. This model was sent as an annex, but it seems the reviewer was unable to access it.

**Reviewer #1:** **Reviewer’s report**

**General Comment**

**This cross-sectional study assessed the impact of the diagnosis of hypertension on self-perceived health status, and attempted to identify some probable factors that may influence this perception. The sources of the data were the National Health Surveys of 2011-2012, 2017 and the 2020 European Health Interview Survey for Spain. From this a total of 66,168 subjects were included. Stratified sampling was performed to get the required subjects from each of these surveys. The statistical analyses that was performed seems adequate. Following the analyses, the researchers concluded that the diagnosis of hypertension and prescription of antihypertensive medication are associated with a lower probability of reporting a “very good” health status, independent of other comorbidities or complications related to the diagnosis. No other factors, including age, were found associated with the perception. Whilst the findings are interesting, they are however not really that novel and do not really add any substantial new information to what we already know. Decreased perception of well-being can be expected with any disease, which may depend on a number of factors, including the level of literacy or level of education of the population, and to some extent the socioeconomic status of the population.**

*W*e sincerely appreciate the comments provided. The impact of a hypertension diagnosis on health status remains unclear. While some studies report a significant impact, others suggest that it only occurs when medication is introduced *(**Trevisol DJ, et al. Health-related quality of life is worse in individuals with hypertension under drug treatment: results of population-based study. J Hum Hypertens. 2012)* or when cardiovascular diseases develop *(Alefishat E, et al Factors affecting health‐related quality of life among hypertensive patients using the EQ‐5D tool. Int J Clin Pract. 2020*). In fact, it has been noted that, as hypertension is an asymptomatic condition, patients often do not perceive it as a disease and may not adhere to medical recommendations *(Katsi V, et al. Arterial hypertension and health-related quality of life. Front Psychiatry. 2017).* We have added information to the introduction section, noting that two studies in our country have reported that, in individuals over 60 years of age, a hypertension diagnosis was not associated with the perception of health status *(Banegas J, et al. Association Between Awareness, Treatment, and Control of Hypertension, and Quality of Life Among Older Adults in Spain. Am J Hypertens. 2006; Forjaz MJ, et al Chronic conditions, disability, and quality of life in older adults with multimorbidity in Spain. Eur J Intern Med. 2015).* The importance of our study lies in demonstrating that the mere diagnosis of hypertension, regardless of whether medication is prescribed or complications arise, affects health perception. We believe that the findings from this representative sample of the Spanish population may shed some light on the ongoing debate.

**Specific comments**

**1. Although the sample size is indeed large, no information on sample size calculation has been included.**

The sample size in each survey wave was determined by its design, estimated to achieve representativeness at the Autonomous Community level. This information, along with the selection criteria and sampling methodology, has been included in the Methods section.

Here’s the modified paragraph:

“Stratified sampling was performed in three phases as part of the survey methodology: first, census sections were selected (2,000 sections in 2011 and 2,500 sections in 2017 and 2020); second, 12 households were chosen from each section in 2011, and 15 households from each section in 2017 and 2020; third, one adult aged 15 years or older was selected from each household. The only selection criteria were being 15 years or older and living in the selected household. All individuals meeting this condition had an equal probability of being selected, but only one person was included from each household. The sample size for each survey was calculated to ensure representativeness at the regional level (Autonomous Community) (29–31)”.

**2. They had indicated the fraction of the population that was considered ‘high class’, but no information was given on how this was derived? Was it based on their income or the locality where they lived or?????**

The classification of social class used in this study is based on a widely recognized proposal that categorizes individuals based on occupation, which is highly correlated with educational background. This proposal includes six categories. Categories I and II, referred to as "High social class" in this study, encompass directors and managers of business establishments, regardless of whether they work in public administration or private enterprise; professionals with university-level education; as well as athletes and artists. Details of the classification can be found in reference 32 (*Domingo-Salvany A, et al. Propuestas de clase social neoweberiana y neomarxista a partir de la Clasificación Nacional de Ocupaciones 2011. Gac Sanit. 2013*). In the Methods section, we explain the occupational categories included in the so-called "High classes."

**3. What fraction was middle class or lower middle class etc?**

We provide a more detailed breakdown of social class in 6 groups for this dataset below, although we believe it may not be necessary in the manuscript as it is merely an adjustment variable. If the editors or reviewers feel otherwise, we have no objection to include the information.

| Group I | 2011 | 2017 | 2020 |
| --- | --- | --- | --- |
| Proportion | 0.109 | 0.112 | 0.116 |
| [95% Conf. Interval] | 0.104- 0.114 | 0.107- 0.118 | 0.111- 0.122 |
|  |  |  |  |
| Group II | 2011 | 2017 | 2020 |
| Proportion | 0.075 | 0.079 | 0.077 |
| [95% Conf. Interval] | 0.071- 0.080 | 0.074- 0.083 | 0.073- 0.082 |
|  |  |  |  |
| Group III | 2011 | 2017 | 2020 |
| Proportion | 0.183 | 0.191 | 0.195 |
| [95% Conf. Interval] | 0.176- 0.189 | 0.185- 0.198 | 0.189- 0.202 |
|  |  |  |  |
| Group IV | 2011 | 2017 | 2020 |
| Proportion | 0.153 | 0.143 | 0.146 |
| [95% Conf. Interval] | 0.147- 0.159 | 0.138- 0.149 | 0.140- 0.152 |
|  |  |  |  |
| Group V | 2011 | 2017 | 2020 |
| Proportion | 0.328 | 0.331 | 0.331 |
| [95% Conf. Interval] | 0.320- 0.336 | 0.324- 0.339 | 0.323- 0.339 |
|  |  |  |  |
| Group VI | 2011 | 2017 | 2020 |
| Proportion | 0.152 | 0.143 | 0.135 |
| [95% Conf. Interval] | 0.146- 0.158 | 0.138- 0.149 | 0.129- 0.141 |

**4. No information has been given on the educational status of the study population. This is important as is most likely going to impact the self-perception. It would have been good if information on the educational status of the study population had been included.**

We agree with the reviewer on the importance of including social characteristics, such as educational level, in health perception studies. It should be noted that in the 2011-12 National Health Survey, the educational level variable was collected for the entire household, not individually, as is the case in the two subsequent surveys. However, our classification of social class, based on occupation, is highly correlated with education. Categories I and II, labeled "High social class," include directors, managers, university-educated professionals, athletes, and artists. All professionals with high education are encompassed within these categories. Given the strong correlation between occupation and educational level, these two variables are typically not used together in models to avoid high collinearity. Therefore, we have included the description of "High social class" in the Methods section to address this issue.

**5. Please include a table showing the results of statistical analyses of the associations between HTN and age and other co-morbidities.**

We are not sure what the reviewer's concern is at this point. The age distribution of hypertension in the Spanish population is well documented in many sources (see, for example, Menéndez E et al . Prevalencia, diagnóstico, tratamiento y control de la hipertensión arterial en España. Resultados del estudio Di@ bet. es. Revista española de cardiología, 2016). Below, we present the percentages of hypertensive individuals within the disease groups studied.

| **Hypertension Status** | **People with CV disease** | **People with Osteoarticular Disease** | **People with Respiratory Disease** | **People with Mental Health Conditions** |
| --- | --- | --- | --- | --- |
| **No** | 43.0 %  (41.4- 44.6%) | 63.0 %  (61.2-64.7%) | 67.6%  (65.3- 69.9%) | 63.2%  (61.2- 65.1%) |
| **Yes** | 57.0 %  (55.5- 58.65%) | 37.0 %  (35.3-38.7%) | 32.4%  (30.1- 34.7%) | 36.7%  (34.9- 38.8%) |

In this other table, the percentages of different conditions within the hypertensive and non-hypertensive groups are shown.

|  | **People with CV disease** | **People with Osteoarticular Disease** | **People with Respiratory Disease** | **People with Mental Health Conditions** |
| --- | --- | --- | --- | --- |
| **No hypertension** | 4.6 %  (4.1- 5.2%) | 18.8 %  (15.9-20.2) | 7.2%  (6.6- 7.9%) | 9.7%  (8.6- 10.9%) |
| **Hypertension** | 22.3 %  (21.1- 23.6%) | 38.4 %  (35.8- 41.1%) | 12.5%  (11.6- 13.5%) | 20.6%  (18.6- 22.6%) |

We are unsure if this is of interest given the study's objectives. Perhaps the reviewer is referring to the need to show the relationships between the adjustment variables and the perception of health status (age, gender, limitation, lifestyles, etc.). We have included all the results of the model in its more interpretable form, the marginal effects, in Appendix 2. Table 3 is a summary of this appendix, featuring variables that not only serve as adjustment variables but also allow for comparing the magnitude of the impact of hypertension on the perception of health status, as proposed in the objective. This way, readers will have all the information available in the appendix as well as the necessary details to follow the main text of the manuscript.

**6. It was interesting to note that those on medication for hypertension were less likely to score a ‘very good’ perception than those who were not taking any medication but knew they had hypertension. What could be the reason for this? No discussion was attempted on this.**

The concept of treatment burden, has been extensively documented in the literature. It refers to the overall workload imposed on patients due to their healthcare regimen, which often results in various negative impacts on their lives. Research has also established a link between this burden and medication adherence *(Zidan A, et al. Medication-Related Burden among Patients with Chronic Disease Conditions: Perspectives of Patients Attending Non-Communicable Disease Clinics in a Primary Healthcare Setting in Qatar. Pharmacy (Basel). 2018).* There are several reasons why a person might have a worse perception of their health status upon receiving a prescription. We can only hypothesize about these reasons. It has been described that inappropriate prescriptions or polypharmacy can worsen the perception of quality of life *(Olsson IN et al . Medication quality and quality of life in the elderly, a cohort study. Health Qual Life Outcomes 2011).* In the case of hypertensive patients, it has already been noted that those receiving pharmacological treatment had a worse perception of their health status compared to those who did not receive such treatment *(Trevisol DJ, et al. Health-related quality of life is worse in individuals with hypertension under drug treatment: results of population-based study Journal of Human Hypertension 2011).* However, it cannot be determined whether this was due to concern about the illness or potential side effects of the medication. We include these ideas in the Discussion section

**7. Why was medication found to be independently associated with the expression of less satisfactory health status? Any speculation?**

Thank you for your suggestion. We have addressed issues 6 and 7 by incorporating the following paragraph:

Among hypertensive patients, those receiving pharmacological treatment often exhibit a worse perception of their health status compared to those not undergoing such treatment, regardless of blood pressure control (20,40). Additionally, the impact of hypertension labeling on health perception may, in part, be attributable to the prescription of antihypertensive drugs (24). This study has corroborated the observed trend. However, after adjusting for the presence of other conditions and polypharmacy, it remains uncertain whether the deterioration in health perception is due to awareness of the condition itself, potential side effects of the antihypertensve medication, or other related factors.

**8. The discussion could be improved in other places too, like e.g. they state that their findings were different from those reported in another survey on the population 15 years earlier but provide no reason or speculation as to why they are different. What was/were the difference/s between these two studies?**

You are right. We have added the following text:

The referenced study focused on individuals over 60 years of age, with an average age exceeding 70, whereas the current research included a sample where 50% of the subjects were under 53 years old. It has been hypothesized that age is linked to a greater acceptance of chronic conditions, particularly those that are asymptomatic, as health expectations adjust throughout life (26). Additionally, since only 64.1% of the hypertensive participants in the previous study were aware of their diagnosis, it is possible that the relationship between a hypertension diagnosis and health perception may be influenced by the labeling effect, as suggested by other studies (40).

**9. They state that there was a total of 66,168 participants, but in Table 1 the total number for smokers and non-smokers is 66085; for body weight it is 61996. What happened to the missing subjects? Please ensure the totals tally and if they don’t then provide a reason.**

The reviewer is correct. Given that this is a population survey, there are some missing responses. We have information on 100% of the subjects for age and gender, 99.87% for smoking habits, and 96.43% for social class. The variable with the least data is BMI, which is only available for approximately 94% of the cases. The final model, which includes only subjects with complete information for all collected variables, is based on 94% of the initial sample. We do not believe the missing data are differential, and therefore, we find that the internal validity of the model is preserved. Nevertheless, any reader can verify the number of missing responses for each variable in the provided database.

**10. They need to emphasise a little more on the relevance and importance of these findings in the discussion. How this will influence the management of hypertension? We know compliance to treatment is one of the major problems in the management of hypertension. Does the perception of the illness impact compliance?**

Thank you for your suggestion. We have revised the paragraph to incorporate these ideas:

The implications of these findings are substantial. Awareness of hypertension is linked to increased adherence to treatment (12,15) but also correlates with a worse perception of health among patients. While disease control does not appear to affect perceived health status, medication is associated with a decline in health perception. Consequently, health professionals, particularly in community settings, should recognize that a hypertension diagnosis can adversely impact patients' health perceptions. Therefore, strategies should focus on both adherence to treatment and enhancing patients' overall coping mechanisms, given that hypertension is a recognized risk factor for cardiovascular disease. These insights are also crucial for health planning and policy development, emphasizing the need to incorporate patients' perspectives into healthcare practices to better address their needs and improve their quality of life.

**11. The title could be revised to be a little more informative**

We proposed this new title:

How a diagnosis of arterial hypertension influences health status perception: findings from a decade-long study in the Spanish population.

**Reviewer #2:** **Reviewer’s report**

**The manuscript titled "Does a diagnosis of arterial hypertension affect the perception of health status? A decade-long study in the Spanish population" is an interesting study, conducted in a community setting, that aimed to assess and discuss the association of being diagnosed with arterial hypertension with self-perceived health status and potential explanative factors.**

**However, the research gap or the novelty of the manuscript is inadequately highlighted, as previous studies had evaluated effects of arterial hypertension on health-related quality of life. This study employed a generalised ordered logit model in its statistical analysis. However, the results and interpretation of the model are not clearly described. In addition, evidence of statistical significance of some of the results described in the manuscript is not available. As a consequence, the discussion and conclusion can only be reviewed objectively and constructively after the authors have addressed the related comments accordingly.**

We appreciate the reviewer's opinion; however, we respectfully disagree. As explained in the introduction, some studies conducted in our country have not found a relationship between a hypertension diagnosis and a decline in quality of life among individuals over 60 years old (*Banegas J, et al . Association Between Awareness, Treatment, and Control of Hypertension, and Quality of Life Among Older Adults in Spain. Am J Hypertens. 2006*). International research suggests that the association between hypertension and poorer quality of life may be more related to the medication (*Trevisol DJ, et al. Health-related quality of life is worse in individuals with hypertension under drug treatment: results of population-based study. J Hum Hypertens. 2012*) or to the cardiovascular diseases develop (*Alefishat E, et al Factors affecting health‐related quality of life among hypertensive patients using the EQ‐5D tool. Int J Clin Pract. 2020*). Additionally, some studies find this association only in women, but not in men (*Kitaoka, M, et al. The relationship between hypertension and health-related quality of life: adjusted by chronic pain, chronic diseases, and life habits in the general middle-aged population in Japan. Environmental Health and Preventive Medicine, 2016*). Finally, other studies clearly identify a negative association between hypertension and health status (*Trevisol DJ, et al. Health-related quality of life and hypertension: A systematic review and meta-analysis of observational studies. J Hypertens. 2011*), though some are based on populations from various regions around the world (*Alefishat E et al . Factors affecting health‐related quality of life among hypertensive patients using the EQ‐5D tool. Int J Clin Pract. 2020; Kaliyaperumal S, et al . Assessment of quality of life in hypertensive patients. J Appl Pharm Sci. 2016*). Therefore, we believe it is important to address this question using data that are representative of the entire Spanish population.

We have incorporated some of these ideas, as detailed in previous responses, into the introduction and discussion to emphasize the necessity and novelty of this study.

Regarding your second concern, the model used was fully detailed in Appendix 1, which was correctly submitted. For reasons beyond the authors' control, it appears that the appendix did not reach the reviewer. However, your comment prompted us to consider the need for additional materials that would facilitate the interpretation of the model's results. Consequently, we have developed Appendix 2, which provides the analysis of the model's marginal effects. These results offer a more direct interpretation, as they illustrate how the probability of being in a specific health status category changes with variations in an independent variable, while holding all other variables at their mean values (we report the marginal effects at means). While Table 3 summarized this appendix, it may be more appropriate to provide the information for all variables included in the model. Additionally, the appendices include the Stata code, allowing for the reproduction of the analysis, as the database is also available.

We now move on to address the comments made by the reviewer in the margins of our manuscript.

**Introduction**

**[The concept of HRQoL overlaps with the self-perception of health status]**

**In what manner(s) does HRQoL overlap with self-perception of health status? Does the overlap contributes to the novelty of the study?**

Thank you for your question. The overlap between Health-Related Quality of Life (HRQoL) and self-perception of health status is indeed discussed in the same paragraph. HRQoL can be understood as the impact of health on an individual's quality of life or as a measure of preference associated with a particular health status. However, the novelty of our study does not stem from this overlap. Instead, it is based on the findings and contributions outlined in the first paragraph of our response to Reviewer 1. We hope this clarification helps.

**[…the awareness of the consequences of suffering the disease and not by the diagnosis itself (20)]**

**How does impact of hypertension on HRQoL contributed by “awareness of the consequences of suffering the disease”, differ from the contribution by the “diagnosis of the disease”? Does the awareness of the consequences of suffering from a disease not typically develop after knowing the diagnosis of the disease? Clearly, this sentence calls for an amendment to render a clear message to readers.**

The effect of a disease on Health-Related Quality of Life (HRQoL) or health perception can begin even before a formal diagnosis, due to symptoms or concerns. In hypertensive patients, the decline in perceived health status often begins not with the hypertension itself—since it is typically asymptomatic—but with the medical diagnosis. Studies have shown that the awareness of having a chronic disease may be responsible for lower quality of life *(Trevisol DJ,et al. Health-related quality of life and hypertension: A systematic review and meta-analysis of observational studies. J Hypertens. 2011).* Therefore, while hypertension itself might not usually directly impact health perception, it could be the medical diagnosis or, in other words, the act of labeling a person with hypertension, that triggers a more substantial negative effect on HRQoL.

We have rewritten the sentence:

However, this impact might be more due to the awareness of the disease's consequences rather than the condition itself.

**[Whether the self-perception of health worsens in hypertensive patients is still unclear, and so is whether this alteration is due to the diagnosis, chronic medication, or comorbidities.]**

**In view that arterial hypertension has been reported to affect HRQoL, it is of immense importance to clearly indicate how this study differs from the previous studies to highlight the novelty of the manuscript.**

We have justified in two paragraphs of the introduction that it is not clear whether the HTN diagnosis worsens self-reported health perception or HRQoL, and that the associations found may be influenced by medication or the presence of other conditions. Our study aims to readdress this question using a representative sample of the general Spanish population, and the findings differ, at least in part, from those reported in previous studies conducted in patients over the age of 60 in our country. If the statement in question is accepted as true, it justifies the objective presented.

**[The aim of this study conducted in the community setting was to analyse the association of HTN with a worse perception of health and determine the potential explanative factors.]**

**Is it necessary to include this word [worse] or will it be better to keep the phrase “perception of health” in a general context?**

Thank you for your suggestion. We believe you are right; we have removed the reference to a worse perception of health and have also reformulated the objective as discussed initially. The revised objective is as follows:

The aim of this study conducted in the community setting was to assess and discuss how a diagnosis of arterial hypertension affects self-perceived health status, examining the association with potential explanatory factors and comparing its impact with that of other chronic conditions.

**Methods**

**[A stratified sampling was performed in three phases: first, selection of census sections (2,000 subjects in 2011 and 2,500 participants in 2017 and 2020); second, selection of 12 households from the 2011 section and 15 from the 2017 and 2020 census sections; third, from each household, an adult >15 years old was selected and a minor, if present (27–29). Only the information on adults was collected from the surveys.]**

**What were the inclusion and exclusion criteria applied in this study?**

**Do provide the reference used to define adult > 15-years-old.**

**The sentence in line 23 is rather confusing- why was a minor included in the sampling (when information was collected from adults)?**

The study is based on secondary data sources, specifically the aforementioned national health surveys. The National Health Survey consists of two modules in each wave: one for adults, which includes individuals aged 15 and older, and one for minors, which covers those under 15. Since only data from the adult module were used in this study, any references to the minor module have been removed to avoid confusion. The inclusion criteria for this study were simple: participants needed to be 15 years or older and reside in one of the selected households. Everyone meeting these criteria had an equal chance of being chosen, though only one individual per household was included in the final sample. There were no additional exclusion criteria, as the primary goal was to ensure that the sample was representative at the regional level. This was achieved through a carefully designed stratified sampling process carried out in three phases.

It should also be noted that, in the Spanish healthcare system, pediatricians attend to the pediatric population up to the age of 14, while patients above this age are seen by general practitioners.

The revised paragraph is as follows:

Stratified sampling was performed in three phases as part of the survey methodology: first, census sections were selected (2,000 sections in 2011 and 2,500 sections in 2017 and 2020); second, 12 households were chosen from each section in 2011, and 15 households from each section in 2017 and 2020; third, one adult aged 15 years or older was selected from each household. The only selection criteria were being 15 years or older and living in the selected household. All individuals meeting this condition had an equal probability of being selected, but only one person was included from each household. The sample size for each survey was calculated to ensure representativeness at the regional level (Autonomous Community)

**[The subject's perception of limitation due to illness (severe, mild, and no limitation, comparing the latter versus the first two) was also included. The number of prescribed chronic drugs other than antihypertensive medication was obtained and polypharmacy was considered present for ≥5 different types of treatment.]**

**1) How was participant’s perception of limitation due to illness defined in the surveys?**

**2) What were the defining criteria for the various categories, i.e., severe, mild and no limitation)? Were standardized criteria applied in all surveys?**

**Do provide reference for the definition of polypharmacy.**

The purpose of these questions was to assess whether a health issue had restricted a person’s ability to engage in their normal activities due to a health problem. This should be assessed according to generally accepted norms regarding the activities that people commonly engage in. The degrees of severity are as follows:

Severely limited

Limited, but not severely

Not limited

The questions used are consistent across all surveys and are as follows:

"During the past six months, to what extent have you been limited in performing activities that people usually do due to a health problem?"

Severely limited

Limited, but not severely

Not limited

Don't know/ No answer

The respondent determines their answer without additional guidance.

All information about the survey questions can be found in references 29 to 31 and on the website:

<https://www.ine.es/dyngs/INEbase/es/operacion.htm?c=Estadistica_C&cid=1254736176783&menu=resultados&idp=1254735573175>

<https://www.ine.es/dyngs/INEbase/es/operacion.htm?c=Estadistica_C&cid=1254736176784&menu=resultados&idp=1254735573175>

We recognize that the numerical definition of polypharmacy offers a simplified perspective on the issue. However, a systematic review of polypharmacy definitions found that the most commonly cited definition was the use of five or more medications daily, as reported in 51 articles, accounting for 46.4% of the reviewed studies (*Masnoon N et al. What is polypharmacy? A systematic review of definitions. BMC Geriatr 2017*).

**Analysis**

**Description of the p-value is not found in the section.**

We decided not to focus on p-values in the Methods section because we support moving away from an overemphasis on statistical significance. Instead of underlininge p-value thresholds, we prefered to integrate a broader context in scientific inference, considering factors such as study design, measurement quality, and external evidence. This approach promotes a more nuanced understanding of the data and helps avoid oversimplified conclusions, fostering a deeper and more thoughtful scientific inquiry that acknowledges the inherent uncertainty and variation in research (*McShane BB et al. Abandon statistical significance. The American Statistician 2019*).

**Results**

**[Of those subjects not diagnosed with HTN, 24.10% and 52.44 % reported a “very good” and “good” perception of their health status, respectively, versus 5.53% and 40.18% of people with HTN. Table 1 shows the characteristics of the overall sample.]**

**It is not clear how the differential percentages were analyzed and whether the differences were statistically significant.**

**Did the cohorts of the various surveys show any statistically significant difference(s)?**

We do not consider that statistical comparisons are necessary in Table 1 or in the description of the sample in line with the STROBE statement that advises against it:

“Inferential measures such as standard errors and confidence intervals should not be used to describe the variability of characteristics, and significance tests should be avoided in descriptive tables. Also, P values are not an appropriate criterion for selecting which confounders to adjust for in analysis; even small differences in a confounder that has a strong effect on the outcome can be important”. *(Vandenbroucke JP, et al. Strengthening the Reporting of Observational Studies in Epidemiology (STROBE): Explanation and Elaboration. PLOS Medicine 2007).*

If the reviewer is interested in knowing whether there are differences in the prevalence of HTN at the three time points, we present the results below, although this is not a primary objective of our study. Additionally, we reiterate that Table 1 has been redone, taking into account the sampling method, so the proportions cannot be calculated without considering the weighting of each response (data shown in the database).

HTN prevalence

2012: 21.29% (CI95%: 19,76- 22.91%)

2017: 22.11% (CI95%: 21.16- 23.08%)

2020: 21.24% (CI95%: 19,89- 22.65%)

**Is this term similar to the term “mild” [limitation] used in page 7; ;line 20 (methods) above? I suggest that the same terminology is used to minimize confusion to readers.**

Thank you for your advice. It has been changed

**[The final model was constructed…]**

**Suggest to illustrate the details of the variables with the aid of a table / tables.**

**Where is the Appendix 1 located? It cant be found in the manuscript.**

**I highly recommend to supplement the description of these findings with a table so as to give a clear picture to the readers.**

We regret once again that Appendix 1 did not reach Reviewer 2, but we assure you that the model was thoroughly documented. After reviewing the entire manuscript in light of some comments, we have decided to add Appendix 2, which reports the marginal effects for each variable. The Methods section and the Results clearly explain the meaning of the coefficients of these marginal effects, specifically the variation in the probability of belonging to a particular category of the dependent variable when the independent variable changes by one unit, while keeping the other variables constant at their means (marginal effects were calculated at the means).

**[Table 2 shows the predicted likelihood of reporting a specific health status for the complete sample and particular subgroups, adjusted for the characteristics of each subject…]**

**[The adjusted likelihood of expressing a “poor” or “very poor” health status did not significantly differ between hypertensive subjects…]**

**Are these results statistically significant? Provide evidence.**

**1) Are the terminologies of “poor” and “very poor” health status referring to “bad” and “very bad” health status respectively? Please ensure that the standardized terms are used throughout the manuscript to minimize confusion.**

**2) Please provide the p value to support the finding.**

**Please indicate the p-values for the relevant results.**

Thank you for your feedback. The terms "poor" and "very poor" have been replaced with "bad" and "very bad" to ensure consistency in the descriptions.

The evidence that the differences are statistically significant is presented in Table 2. The model-adjusted probability of reporting a "very good" health status in the hypertensive population is 17.69% (95% CI: 14.88-20.51%), and in the treated hypertensive population, it is 14.29% (95% CI: 12.55-16.03%). Since these probabilities in the non-hypertensive population are 23.57% (95% CI: 21.76-25.38%) and the confidence intervals do not overlap, we can say with a high level of confidence (95%) that hypertensive and treated hypertensive populations are less likely to report a "very good" health status than the non-hypertensive population. In the case of "bad" and "very bad" health status, the confidence intervals do overlap, so we cannot say that the differences are statistically significant.

We respectfully disagree with Reviewer 2's suggestion regarding the importance of reporting p-values. Gardner and Altman, in their BMJ article, argued that "the excessive use of hypothesis testing at the expense of more informative approaches to data interpretation is an unsatisfactory way of assessing and presenting statistical findings from medical studies." They advocate for the use of confidence intervals, stating that these "present the results directly on the scale of data measurement" and should "become the standard method for presenting the statistical results of major findings." *(Gardner MJ and Altman DG. Confidence intervals rather than P values: estimation rather than hypothesis testing. BMJ 1986*). This perspective aligns with our approach and is why we have chosen not to emphasize p-values in our analysis. In 2016, the American Statistical Association stated: "In view of the prevalent misuses of and misconceptions concerning p-values, some statisticians prefer to replace p-values with other approaches. These include methods that emphasize estimation over testing, such as confidence, credibility, or prediction intervals [...]; All these measures and approaches rely on further assumptions, but they may more directly address the size of an effect (and its associated uncertainty) or whether the hypothesis is correct" *(Wasserstein RL and Lazar NA. The ASA's Statement on p-Values: Context, Process, and Purpose, The American Statistician, 2016).*

In our case, we have chosen to report confidence intervals for the probabilities predicted by the model and for the calculations of marginal effects, as these provide an idea of the magnitude of the association, which p-values do not. However, the full model can be seen in Appendix 1, and the marginal effects in Appendix 2, which include p-values for coefficients and marginal effects.

**[Table 3 displays the percentage differences…]**

**Where can Appendix 1 be found?**

**Which statistical methods were employed to obtain these findings? They need to be mentioned in the statistical analysis above.**

We have already explained what we believe has happened with Appendix 1. The results in Table 3 represent the marginal effects of the variables listed (along with their 95% confidence intervals). The meaning of the marginal effects has been explained in both the Methods and Results sections. Appendix 2 presents the marginal effects for the entire model.

**[*: do not reach statistical significance set at p<0.05.]**

**This statement regarding statistical significance is not written in the typical style as that commonly found in any publications. Any reasons for having it written in such a manner?**

We agree with the reviewer. It does not make sense to discuss p-values here; we merely wanted to visually indicate that the confidence interval included the value 0, and therefore the difference was not statistically significant. We have removed the reference.

**Discussion**

**This section lacks discussion on the data obtained from the generalised ordered logit model used in the study which should form the major bulk of the discussion. The variables used in the model and their related information could possibly be presented in Annex 1 as mentioned in the section of Results above. Unfortunately, the Annex 1 appears to be missing in this manuscript.**

**In addition, the results discussed in this section need to be addressed accordingly as highlighted in their respective areas in the results section and they are mostly pertaining to statistical significance. Until these are addressed, reviewing of the associated discussions cannot be conducted objectively and constructively.**

**At this point in time, objective and constructive comments on the conclusions cannot be made until the above comments for the results and discussion are addressed.**

We sincerely regret the difficulties encountered by Reviewer 2; however, we must respectfully reiterate that these issues are beyond the authors' responsibility. While we appreciate all the comments made, we disagree that the importance of this manuscript rests on the statistical significance of its results. What we emphasize is the magnitude of the associations found (which, of course, are statistically significant). The impact of HTN diagnosis on the likelihood of reporting a "very good" health status is substantial and is compared with the impact of other chronic conditions known to affect health perception, such as anxiety/depression or musculoskeletal disease. Following the review recommendations, this section has been improved by discussing the differences found with other studies conducted in the country and examining the potential importance of antihypertensive medication, in addition to diagnostic labeling, on health perception. Additionally, the paragraph concerning the implications of the research has been expanded. We believe that the contributions made in the review have also helped improve this section.

We appreciate all the comments provided, as we believe they will enhance the quality of the manuscript. We hope that this revised version meets the standards for publication, but we remain open to reviewing any additional points you feel require further modification.

Sincerely,

Dr. Jesús Martín-Fernández

On behalf of the authors
