# Supplementary material for: Association between hypertension and self-perception of health status: Findings from a decade population-based survey in Spanish adults
Source: PLoS One. 2025 May 7;20(5):e0322577. doi: 10.1371/journal.pone.0322577 (PMC12057956; doi:10.1371/journal.pone.0322577)
Supplement: Attachment — Submitted filename: To the Editorial Team_rev2_def.docx [file pone.0322577.s008.docx]

To the Editorial Team:

We sincerely appreciate the opportunity to revise and resubmit our manuscript, formerly titled “How a diagnosis of arterial hypertension influences health status perception: findings from a decade-long study in the Spanish population,” as well as the dedication of the editorial team and reviewers in providing valuable feedback to improve our work.

We understand the concern raised regarding the perceived lack of novelty and would like to emphasize the unique contribution of our study. Our research is based on data from a nationally representative survey with a robust methodological design, conducted over more than ten years. The scale and representativeness of this dataset provide a comprehensive and reliable foundation to explore how the diagnosis of hypertension impacts health status perception. These results offer valuable insights that are directly applicable to public health policy and clinical practice, addressing a gap in the understanding of this relationship within large and diverse populations.

We are committed to addressing the concerns about novelty and clearly highlighting the knowledge our study adds to fill existing research gaps in the revised manuscript. We are confident that these enhancements will further enrich the manuscript.

**ACADEMIC EDITOR:**

**Although one reviewer has recommended accept for publication. However, another reviewer, which has the same opinion with Reviewer#2, raised concern by the lack of novelty of this work, as there are multiple works similar in nature being published previously. As much as the work is scientifically valid, what are the new knowledge / information that would fill the current research gap is essential. I suggest the authors to relook this matter, and highlight the novelty / new knowledge that would narrow the research gap.**

We acknowledge that the relationship between hypertension and health perception has been explored in diverse contexts and through various methods. However, this link remains not fully understood, with studies often producing contradictory findings.

In 2008 a study analyzing HRQoL among 8,303 adults from the 2001–2004 NHANES cohort found that individuals with hypertension reported poorer health outcomes, including fair or poor health status, more unhealthy days, and increased activity limitations. Those aware of their hypertension had even worse HRQoL, with more physically unhealthy days and mental health challenges. Antihypertensive medication was linked to more physically unhealthy days, but HRQoL did not differ significantly based on blood pressure control status (Hayes et al., 2008). However, the study did not examine the impact of comorbidities on these outcomes.

In 2011, Trevisol et al., in a systematic review of over 20 studies, concluded that the health perception of individuals with hypertension is slightly worse than that of normotensive individuals. The review emphasized that the influence of high blood pressure and the awareness of having hypertension require further investigation (Trevisol et al., 2011).

One year later, the same author conducted a population-based study with nearly 2,000 patients and concluded that the worse health perception among individuals with hypertension is more evident in patients undergoing treatment, particularly when their blood pressure is controlled by medication (Trevisol et al., 2012).

Other studies have explored the role of comorbidities in reducing health perception attributed to hypertension, finding that hypertension itself significantly reduces health perception. This impact is further influenced by various factors, such as gender, education level, blood pressure control, and physical exercise. Numerous studies have also highlighted a strong association between drug-related factors—such as the number of prescribed medications, disease duration and severity, and the presence of adverse drug reactions or complications—and diminished health perception (Shah et al., 2020).

In contrast, Katsi et al. (Katsi et al., 2017), in an observational study of a European population, concluded that the stage of hypertension and awareness of the disease do not significantly affect patients' physical or mental health. This finding suggests that the asymptomatic nature of hypertension in its early stages may explain the lack of impact on health perception.

Moreover, some studies have shown that health perception in hypertensive patients can differ based on personal, regional, and contextual factors. For example, differences in health perception have been observed between urban and rural populations, with factors such as education, marital status, employment, health behaviors, and access to healthcare influencing these differences (Zhang et al., 2016). This highlights the importance of considering both individual and contextual factors when investigating health status perception.

Therefore, it is particularly relevant to examine the impact of hypertension within the specific social and cultural context of Spain. Understanding how the disease is perceived and managed in this unique context is essential for developing targeted interventions that address the particular needs and circumstances of the population. This approach will provide a more accurate and culturally sensitive understanding of how hypertension influences health perception.

In evaluating the published evidence in Spain, it becomes evident that the relationship between hypertension and health perception is complex and not fully understood. While hypertension is a well-established risk factor for various health complications, its direct impact on individuals' perception of their health appears limited when it occurs without comorbidities.

For instance, a study conducted in Spain in 2006, involving 3,368 participants aged 60 or older, used the SF-36 questionnaire to assess HRQL. The study found no significant association between hypertension, antihypertensive treatment, or blood pressure control and worse HRQL in the overall sample. These findings suggest that, in the absence of additional health conditions, hypertension does not directly deteriorate health perception (Banegas et al., 2006).

Similarly, Forjaz et al. analyzed chronic conditions, disability, and quality of life among older adults with multimorbidity in Spain. They concluded that, although hypertension was highly prevalent, it was not significantly associated with worse quality of life (Forjaz et al., 2015).

Further supporting this perspective, a population based study of over 11,000 individuals examined the joint impact of nutritional status and chronic diseases on self-rated health. Among participants with normal weight, hypertension alone was not associated with a higher probability of reporting poor health status. However, when hypertension coexisted with overweight or obesity, the likelihood of reporting poor health status increased substantially, particularly for those with additional chronic conditions such as diabetes (Sayón-Orea et al., 2018). These results reinforce the idea that hypertension in isolation has a limited impact on health perception, whereas the combination of hypertension with other health challenges significantly amplifies the likelihood of reporting poorer health.

In light of these findings, we argue that the relationship between hypertension and health perception cannot be generalized across all populations and contexts. It is essential to consider the broader social, cultural, and individual factors that influence this relationship. The present study seeks to evaluate the impact of a hypertension diagnosis on health perception, while carefully isolating the effects of medication, comorbidities, and other circumstances known to influence this perception. This approach addresses critical gaps in understanding how the diagnosis itself shapes health perception independently of confounding factors.

Furthermore, our study compares the impact of hypertension on health perception with that of other chronic diseases, which provides valuable context for understanding the magnitude of hypertension's impact on health perception. By leveraging the most representative population health data source available in our context, constructed with a robust and reliable methodology, the study ensures that the findings are grounded in high-quality evidence. This makes the findings highly relevant to clinical and public health discussions.

By addressing these gaps, the study contributes to a nuanced understanding of how hypertension affects individuals' health perception, laying the groundwork for tailored interventions aimed at improving outcomes in hypertensive patients. We believe this justifies the pertinence of conducting this research.

Some sentences in the introduction have been rewritten to reinforce the pertinence of the study (see the version with tracked changes).

The section on the implications of the study has been rewritten to highlight the contribution of this work to the current state of knowledge.

**Reviewer #1:** **Reviewer’s report**

**Thank you very much for asking me to review the revised manuscript titled ‘How a diagnosis of arterial hypertension influences health status perception: findings from a one decade-long study in the Spanish population’. My comments are appended below**

**Overall Comment**

**The authors have made a substantial effort in addressing the reviewers’ comments. I am satisfied with the revisions and their rebuttal. However, you may want to re-look at the following before accepting it for publication.**

Thank you for your feedback. We appreciate your comments and will review the remaining points carefully.

**Minor comments**

1. **It will be useful to the readership if the fractions of middle or lower middle class of the study population are also included.**

The weighted percentages of middle and lower social classes have been incorporated into Table 1.

2. **I still think it is only appropriate for them to just state some of the reasons for the differences in the number of participants in the initial and final analysis. I realise there are always going to be incomplete data entries by participants when dealing with population surveys, but it is, nevertheless, appropriate to state the reasons briefly, instead of expecting the readers to refer to the database for reasons of the differences in the initial and final study population.**

The final model was built with 61,933 observations, representing the number of subjects for whom all variables included in the model were available. Missing values resulted from either the interviewed subject not providing the data or losses and errors during data collection and management. No imputation method was used for missing data.

The information provided in the next paragraph is included in the Results section to address your suggestion. This ensures that readers can understand the reasons for the differences in the number of participants between the initial and final analysis without needing to refer to the database.:

“The initial sample included 66,168 subjects: 21,007 in 2011, 23,089 in 2017, and 22,072 in 2020. Demographic and health status data were available for all subjects. Information on social class was collected for 63,805 subjects, BMI for 61,996, and marital status for 66,043. Of the total, 66,120 provided details on physical activity, and 66,085 on smoking habits”.

3. **With reference to the title, it could be revised further in my opinion. The study data presented is not indicating about the ’how’ or the ‘why’ the perception of health is the way it is. It is rather documenting an association between the diagnosis and treatment of hypertension and the probability of negative perception of their health status. Now, it is not wrong to have a title that clearly includes the main message arising from the study itself. They may, therefore, wish to consider the following title - “Diagnosis of hypertension and antihypertensive prescription is associated with a negative self-perception of health status”. It is what they have concluded from the study.**

We appreciate your suggestion and have carefully reviewed the title in light of your feedback. However, after consideration, we are not fully convinced that incorporating the study's main result directly into the title is the best approach. Instead, we believe a broader title better captures the scope of the research. Following your input, we have decided on the following title: " Association between hypertension and self-perception of health status: Findings from a decade population-based survey in Spanish adults "

4. **The word ‘data’ is a plural word, and they will need to revise the words ‘data was’ to ‘data were’ in a couple of places in the manuscript.**

Thank you for your suggestion. We have made the correction**.**

**Reviewer #3: Reviewer’s report**

**There have been several previous reports on the perception of health among patients with hypertension which therefore lacks the novelty of this report.**

We appreciate the reviewer's comment, but as mentioned in our response to the editor, we hold a different opinion. While we acknowledge that the relationship between hypertension and health perception has been explored in various contexts, it remains incompletely understood, with many studies yielding contradictory results. For instance, research has shown that hypertension alone may not always lead to worse health perception, particularly when no comorbidities are present. Furthermore, studies have indicated that factors such as gender, education, medication use, and comorbid conditions can significantly influence how individuals perceive their health. However, research on this topic remains fragmented, with varying conclusions based on the context and population studied. Our study aims to address these gaps, offering a more comprehensive understanding of the impact of hypertension diagnosis on health perception within the specific social and cultural context of Spain. This focused approach, isolating medication, comorbidities, and other relevant factors, is intended to provide clearer insights into how hypertension itself influences health perception. We believe our study fills an important gap in the existing literature and justifies the relevance of this research.

Both the introduction and the discussion have been updated with new paragraphs to emphasize the contribution of this study to the knowledge on the relationship between hypertension and health perception in our context.

We greatly appreciate all the comments and suggestions provided, as we believe they will contribute to improving the quality of the manuscript. We hope that this revised version meets the necessary standards for publication, and we remain open to addressing any additional points you feel may require further revision.

Sincerely,

Dr. Jesús Martín-Fernández

On behalf of the authors
